# Fully recyclable multifunctional adhesive with high durability, transparency, flame retardancy, and harsh-environment resistance

**DOI:** 10.1126/sciadv.add8527

**Published:** 2022-12-14

**Authors:** Zi-Hao Wang, Bo-Wen Liu, Fu-Rong Zeng, Xin-Cen Lin, Jia-Yan Zhang, Xiu-Li Wang, Yu-Zhong Wang, Hai-Bo Zhao

**Affiliations:** Collaborative Innovation Center for Eco-Friendly and Fire-Safety Polymeric Materials (MoE), State Key Laboratory of Polymer Materials Engineering, National Engineering Laboratory for Eco-Friendly Polymer Materials (Sichuan), College of Chemistry, Sichuan University, Chengdu 610064, P.R. China.

## Abstract

Recyclable/reversible adhesives have attracted growing attention for sustainability and intelligence but suffer from low adhesion strength and poor durability in complex conditions. Here, we demonstrate an aromatic siloxane adhesive that exploits stimuli-responsive reversible assembly driven by π-π stacking, allowing for elimination and activation of interfacial interactions via infiltration-volatilization of ethanol. The robust cohesive energy from water-insensitive siloxane assembly enables durable strong adhesion (3.5 MPa shear strength on glasses) on diverse surfaces. Long-term adhesion performances are realized in underwater, salt, and acid/alkali solutions (pH 1-14) and at low/high temperatures (−10-90°C). With reversible assembly/disassembly, the adhesive is closed-loop recycled (~100%) and reused over 100 times without adhesion loss. Furthermore, the adhesive has unique combinations of high transparency (~98% in the visible light region of 400-800 nm) and flame retardancy. The experiments and theoretical calculations reveal the corresponding mechanism at the molecular level. This π-π stacking–driven siloxane assembly strategy opens up an avenue for high-performance adhesives with circular life and multifunctional integration.

## Teaser

Multifunctional adhesives are endowed with circular life and durability under harsh conditions.

## INTRODUCTION

Adhesives, as important and essential materials, have been widely used in daily life and industry (*1*–*5*). The demand for adhesives has grown substantially over the past few decades. In 2021, China’s adhesive production reached 7.09 million tons. However, the adhesives used in large quantities end up as garbage, causing serious environmental problems and waste of resources (*6*). In particular, because of the stable cross-linked structure and strong interfacial interaction, conventional commercial adhesives, such as epoxy and polyurethane resins (*7*, *8*), are difficult to remove from the surface of the bonded substrate after the end of their service life. Consequently, the disposal of massive waste adhesives and their binding substances will be a headache for the future (*9*). There is an urgent need to develop sustainable adhesives that can be recycled without complex handling.

Developing reversible adhesives that allow full recycling is an attractive strategy for their sustainability (*10*). Recently, several advances have been made in the preparation of reversible adhesives, including the introduction of stimuli-responsive functional groups (e.g., temperature and photoisomerization groups) (*11*–*15*), dynamic covalent bonding (e.g., disulfide and Diels-Alder bonds) (*16*–*18*), and dynamic noncovalent interactions (e.g., supramolecular interactions and hydrogen bonds) into the molecular structure (*19*–*21*). For instance, azobenzene derivatives can be used to fabricate reversible adhesives via phase transitions triggered by green light (*13*). In another study, the designed ionogel adhesive exhibited reversible and reusable properties because of interfacial supramolecular interactions and well-tuned viscoelasticity (*22*). Despite much progress, however, most of the reported reversible adhesives suffer from low bond strength and poor durability, especially under harsh conditions, seriously limiting their long-term applications in high-humidity, underwater, or cold/hot environments (*23*, *24*). A major challenge in adhesion science is to design and prepare completely recyclable adhesives with robust and durable adhesion performances in harsh environments.

In addition, multifunctionality, such as transparency and flame retardancy, is a key requirement for reversible adhesives in many specific applications. For example, bonding of devices for optical applications often needs to maintain high transparency (*25*, *26*), while adhesives for electronic device applications often require high flame retardancy to reduce potential fire risks (*27*). Unfortunately, there are few reports on reversible adhesives with multifunction, probably because of the lack of suitable material design and construction methods. Therefore, reversible/recyclable adhesives with robust adhesion strength, excellent durability, high transparency, and flame retardancy are appealing and have the potential to make great contributions in various fields.

Poly(organosiloxane)-based materials, characterized by antiaging properties, low surface energy, and moist heat tolerance (*28*), may be a preferential choice for multifunctional adhesives with harsh-condition resistance. Conventional silicone glues are still difficult to reverse because of the covalently cross-linked network of Si─O─Si with high bond energy (534 kJ mol^−1^) (*29*). Taking advantage of multiple noncovalent interactions, poly(organosiloxane)-based supramolecular materials with reversible cross-linking have been developed with unique properties such as intrinsic healing, stimuli responsiveness, and transparency, which are promising in artificial skin (*30*, *31*), as well as advanced coatings (*32*–*34*). However, in terms of adhesive applications, current reversible poly(organosiloxane)-based materials are hindered by unsatisfactory bonding strength and weak underwater adhesion (*35*, *36*). How to construct reversible poly(organosiloxane)-based adhesives with specific conditional responses for closed-loop cycling under mild conditions is still unknown.

Here, we demonstrate the development of reversible multifunctional poly(organosiloxane)-based adhesives with important but rarely reported combined advantages including completely closed-loop recycling, strong underwater bonding strength, high durability, transparency, and resistance to harsh environments. In our scheme, a P-doped aromatic siloxane derivative is designed to prepare the multifunctional adhesive that can reversibly self-assemble in an orderly manner on the substrate surface driven by π-π stacking. The resulting adhesive shows strong and durable adhesion (up to 3.5 MPa) on various materials because of the robust cohesive energy caused by water-insensitive π-π interactions. Even in harsh conditions of salt/acid/base solutions (pH 1 to 14) and a wide temperature range (−10° to 90°C), the strong adhesion effect can be maintained for a long time without decay. The adhesives can be completely closed-loop recycled and reused more than 100 times, which is realized by the repeated assembly/disassembly of adhesive through simple infiltration-volatilization in ethanol. In addition, the adhesive has high transparency with 98% light transmittance and excellent flame retardancy. This work will greatly inspire the next generation of sustainable adhesives with full recyclability and multifunctionality.

## RESULTS

### Preparation and characterization

**Fig. 1.**
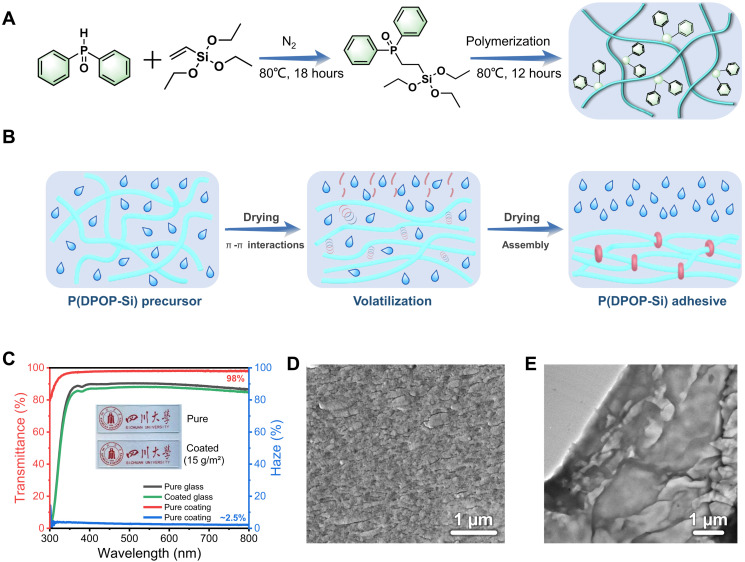
Preparation and assembly of P(DPOP-Si) adhesive. (**A**) Synthesis route of DPOP-Si and P(DPOP-Si). (**B**) Illustration of the assembly process of P(DPOP-Si) driven by π-π interactions during ethanol volatilization. (**C**) Transparency of P(DPOP-Si) adhesive. (**D** and **E**) Cross-sectional SEM and TEM image for P(DPOP-Si) adhesive.

In the design of the adhesive structure, low surface energy poly(organosiloxane) is used as a durable and water-resistant cross-linked backbone, while phosphorus-containing aromatic pendant groups provide π-π interaction sites for further self-assembly. As shown in Fig. 1A, we started with the synthesis of the functional monomers (DPOP-Si) from a one-step facile addition reaction between triethoxyvinylsilane (VTES) and diphenylphosphine oxide (DPOP) (*37*). Subsequently, the assembly precursor of the multifunctional adhesive [P(DPOP-Si)] was obtained by the hydrolysis condensation reaction of DPOP-Si in an ethanol/water medium with overall yields of >90%. Their chemical structures were confirmed by ^1^H nuclear magnetic resonance (NMR) (fig. S1) and Fourier transform infrared (FTIR) (fig. S2) spectra, and more detailed analyses are listed in the Supplementary Materials. Last, the P(DPOP-Si) precursor with a relatively low degree of condensation can be dissolved in ethanol to form nanomicelles (~4 nm; fig. S3), which, in turn, are used as binders for various substrates. During further ethanol volatilization, P(DPOP-Si) nanomicelles assemble through π-π interactions, providing strong cohesive energy (Fig. 1B). The corresponding mechanism in detail will be described in the “Mechanism for reversible strong adhesion” section.

The resultant P(DPOP-Si) adhesive is colorless and highly transparent. As observed in Fig. 1C (inset digital picture) and fig. S4A, the glass loaded with ~400- to 500-μm-thick adhesives showed equivalent transparency to the pristine one in naked eye vision. After loading with P(DPOP-Si), the transmittance of the glass only slightly decreased in the visible wavelength range of 400 to 800 nm. Note that, excluding the influence of glass, the P(DPOP-Si) adhesive exhibited a light transmittance as high as 98% and a low haze of only ~2.5%, much superior to previously reported values (table S2) (*13*, *15*, *23*, *36*, *38*). This high transparency is probably due to the ordered assembly of P(DPOP-Si). The top surface (fig. S4B) of the adhesive was smooth without phase separation, while its side cross section (Fig. 1D) showed an ordered, layered, and stacked microstructure with a thickness of 100 to 300 nm (*39*). This layer-by-layer assembly structure was also confirmed by transmission electron microscopy (TEM) imaging (Fig. 1E). It was speculated that ethanol volatilization induced the layered assembly of P(DPOP-Si) driven by directional π-π stacking, which further contributed to the strong, durable, and reversible adhesion of P(DPOP-Si).

### Strong and durable adhesion performance

**Fig. 2.**
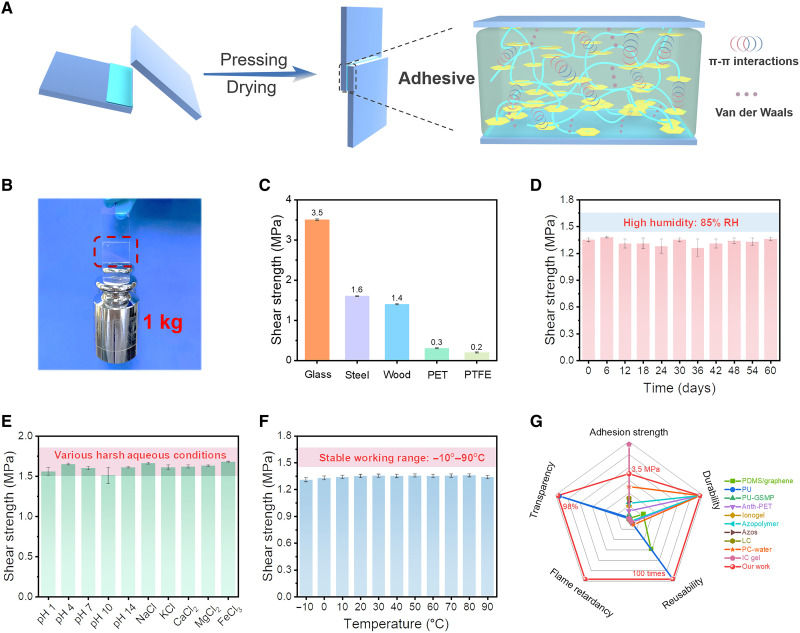
Adhesion properties for different substrates and durable adhesion under various harsh environments. (**A**) Adhesion procedure of P(DPOP-Si) with π-π and van der Waals interactions. (**B**) Photograph of P(DPOP-Si) adhesive on glass surfaces holding a 1-kg weight. (**C**) Shear adhesion strength of P(DPOP-Si) adhesive on various substrate surfaces (glass, steel, wood, PET, and PTFE). (**D**) Adhesion stability of P(DPOP-Si) adhesive in a high-humidity environment of 85% RH for 60 days. (**E**) Adhesion stability of P(DPOP-Si) adhesive soaked in various solutions with pH values of 1 to 14 and different salt solutions (NaCl, KCl, CaCl_2_, MgCl_2_, and FeCl_3_) for 7 days. (**F**) Relationship between the shear adhesion strength and working temperature of the adhesive. (**G**) Comparison of P(DPOP-Si) adhesive with other reported reversible adhesives in terms of shear adhesion strength, durability, transparency, reusability, and flame retardancy.

Following the procedure in Fig. 2A, the adhesion performances between various separated substrates bonded by the P(DPOP-Si) adhesive were monitored in detail (*40*). Note that the adhesive with a small contact area (about 2 cm × 1 cm) on the smooth glass substrate could hold a 1-kg weight easily (Fig. 2B). The robust microscopic adhesion performance of the P(DPOP-Si) adhesive is attributed to the π-π and van der Waals interactions existing among the adhesive molecules and the adhesive/substrate interface (Fig. 2A) (*36*). The P(DPOP-Si) adhesive was almost invisible because of the high transparency, resulting in “markless” bonding. The quantitative shear adhesion strength of the adhesive for several representative substrates, including glass, steel, wood, polyethylene terephthalate (PET), and polytetrafluoroethylene (PTFE), was determined. As shown in Fig. 2C and fig. S5, the adhesive exhibited high adhesion on different substrates in the following order: glass > steel > wood > PET > PTFE. In addition to the cohesive energy of the adhesive itself, the adhesion strength is highly related to the interaction/compatibility at the adhesive/substrate interface. The P(DPOP-Si) adhesive shows the highest shear adhesion strength (3.5 MPa) on glass surfaces because of the strong intermolecular interactions and the good compatibility between Si-rich interfaces (*41*). As for steel and wood, the adhesion strength is reduced to 1.6 and 1.4 MPa. Even for PTFE with an inert surface where fewer hydrogen bonding and hydrophobic interactions existed, the adhesion strength achieved 200 kPa (*15*, *36*). The P(DPOP-Si) adhesive shows high transparency and broad strong adhesion to various substrates, comparable to most adhesives reported under normal conditions.

The P(DPOP-Si) adhesive displays excellent long-term durability in a variety of harsh conditions. As observed in Fig. 2D, the P(DPOP-Si) adhesive maintained stable adhesion on the wood surface without pronounced decay after being placed in a high-humidity environment of 85% relative humidity (RH) for 60 days. On account of the practical applications in different aqueous environments, the adhesion performances of the P(DPOP-Si) adhesive immersed in different ionic salt and pH solutions were further evaluated. It is known that common reversible adhesives typically weaken hydrogen bonding/electrostatic interactions in high ionic strength and acid/base environments (*42*, *43*). The adhesion strength of P(DPOP-Si) bonded to steel substrates remained stable (~1.6 MPa) during the 1-week immersion in different salt solutions such as NaCl, KCl, CaCl_2_, MgCl_2_, and FeCl_3_. Even after one weak immersion in solutions with pH values ranging from 1 to 14, the bonded steel substrate still maintained the original strong adhesion. The excellent durability to different harsh water environments can be attributed to the stable self-assembly of P(DPOP-Si) driven by water-insensitive π-π stacking. In addition, most of the reported reversible adhesives may fail under high/low-temperature conditions, which will greatly limit their practical applications. However, the P(DPOP-Si) adhesive can work stably with strong adhesion over a wide temperature range (−10° to 90°C), which is conducive to long-term use in hot/cold environments (Fig. 2F). Beyond the working temperature range, the π-π stacking interactions of P(DPOP-Si) are disrupted, resulting in a decrease in bond strength. To further reveal the stability of the P(DPOP-Si) adhesive against different chemical environments, dissolution/micelle size experiments with a variety of different typical solvents were performed. As shown in fig. S6, P(DPOP-Si) rapidly transformed into homogeneous nanomicelles in strong polar solvents such as butanol, hexanol, acetone, tetrahydrofuran (THF), dimethyl sulfoxide (DMSO), and trichloromethane (TCM). Moreover, the diameters of the nanomicelles were in the range of 2 to 30 nm. In contrast, similar to water resistance, the P(DPOP-Si) binder was completely insoluble in low-polarity organic solvents [toluene, *n*-hexane, and petroleum ether (PE)] and exhibited excellent resistance. This phenomenon can be attributed to the cohesive energy of water-insensitive siloxane assemblies driven by π-π stacking, which is stable for low-polarity organic solvents but not for strong-polarity organic solvents. Consequently, the P(DPOP-Si) adhesive exhibits high durability in water, toluene, *n*-hexane, and PE and can be recycled with the help of strong polar organic solvents. The excellent resistance to harsh conditions of high humidity, underwater and salt/acid/alkali solutions, and low/high temperatures enables the P(DPOP-Si) adhesive to handle complex application environments.

### Completely reversible/recyclable performance

**Fig. 3.**
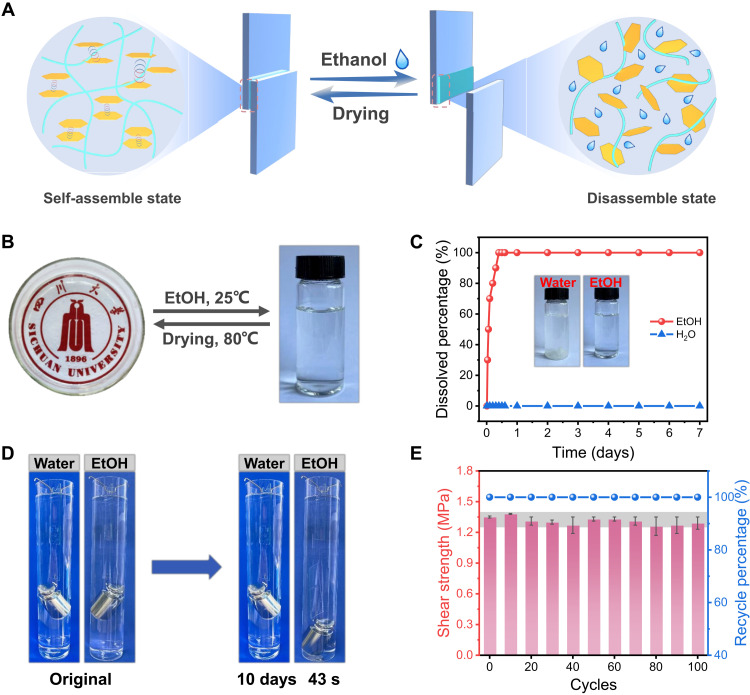
Reversible assembly and reusability of P(DPOP-Si) adhesive. (**A**) Illustration of the reversible transformation between the assembly state and the disassembly state for P(DPOP-Si) adhesive. (**B**) Macroscopic process of the infiltration-volatilization cycle for P(DPOP-Si) adhesive in ethanol. (**C**) Stability of P(DPOP-Si) adhesive in ethanol and water for different times. (**D**) Macroscopic test of adhesion behavior for ethanol response. (**E**) Adhesion strength of P(DPOP-Si) adhesive after different reused cycles.

The reversibility/recyclability of adhesives is a fascinating feature to effectively alleviate serious environmental problems and waste of resources (*44*). Despite strong and durable adhesion in water or even under harsh conditions, we found that the P(DPOP-Si) adhesive could be easily removed from substrates by simply rinsing or soaking in ethanol solvents along with the disassembly process (Fig. 3A). This result suggests that P(DPOP-Si) is applicable as a completely reversible/recyclable underwater adhesive that can be reused repeatedly through ethanol-induced reversible self-assembly/disassembly. As described in Fig. 3B, the P(DPOP-Si) adhesive loaded in a petri dish could be freely infiltrated by ethanol at ambient temperature within a short time, then transformed into homogeneous colorless and transparent nanomicelles, and completely re-adhered on the dish after ethanol volatilization. To further verify the special infiltration in ethanol but excellent durability against water, the P(DPOP-Si) adhesive was directly incubated in ethanol and water for 7 days (Fig. 3C). In ethanol medium, the P(DPOP-Si) adhesive was dissociated rapidly to form a homogeneous solution with a 100% percentage. In contrast to the water medium, the dissolved percentage of the P(DPOP-Si) adhesive nearly remained unchanged (down to 0) after 7 days of immersion.

For macroscopic adhesion, the P(DPOP-Si) adhesive also exhibited both rapid reversibility/recyclability and excellent durability. As shown in Fig. 3D, two separated glass substrates bonded by the P(DPOP-Si) adhesive were stable enough to carry a weight of 200 g when immersed in water for 10 days. In contrast, after immersion in ethanol, it fell off quickly within 43 s due to transformation to a dissolved state, exhibiting sensitive responsiveness. Similarly, the P(DPOP-Si) adhesive can be completely recycled and repeatedly reused on a variety of substrates. We further evaluated the recycling-reusing stability of P(DPOP-Si) by reversibly adhering to rough wood surfaces, where the adhesive is relatively difficult to recycle. Figure 3E and fig. S7 show that the adhesive strength remained stable without obvious decay during the 100 cycles of adhesion. In particular, it was able to maintain an ideal recycling rate of up to 100% in perpetuity, revealing the characteristics of complete closed-loop recycling of the P(DPOP-Si) adhesive. The whole recycling process did not use toxic solvents and high energy consumption. This green and facile recyclable strategy is highly beneficial to the sustainable development of adhesives. While maintaining complete reversibility and recyclability, the P(DPOP-Si) adhesive exhibits great combined advantages of high adhesion strength, durability, robust tolerance toward various harsh conditions, transparency, and flame retardancy, far superior to previous reports (Fig. 2G and table S2).

### Mechanism for reversible strong adhesion

**Fig. 4.**
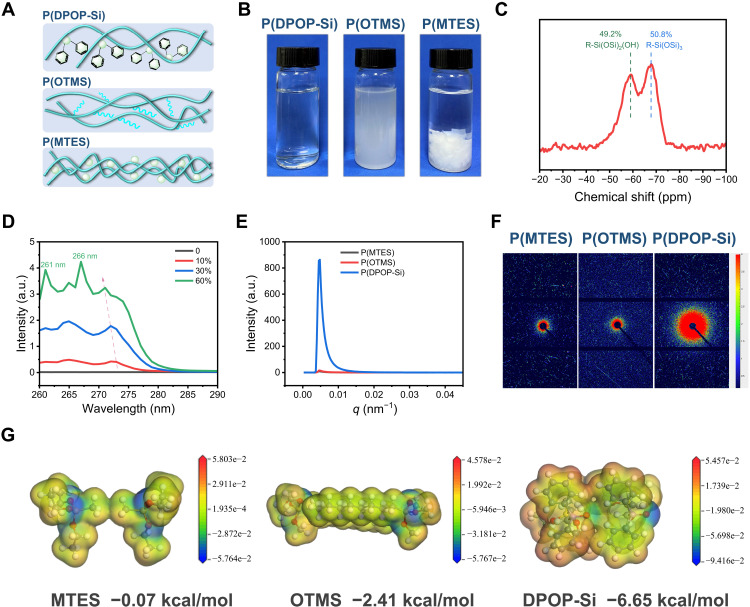
Mechanism of reversible assembly/disassembly for P(DPOP-Si) adhesive. (**A**) Illustration of the polymer networks for P(DPOP-Si), P(OTMS), and P(MTES). (**B**) Dissolving behaviors of P(DPOP-Si), P(OTMS), and P(MTES) in ethanol. (**C**) ^29^Si magic angle spinning NMR spectra of the P(DPOP-Si) network. ppm, parts per million. (**D**) UV/Vis spectra of P(DPOP-Si)/ethanol solutions with different concentrations. a.u., arbitrary units. (**E** and **F**) One-dimensional (1D) SAXS integral curves and 2D SAXS patterns for P(MTES), P(OTMS), and P(DPOP-Si). (**G**) Energy distribution mappings for MTES, OTMS, and DPOP-Si through DFT calculations.

Here, we elucidate the unique mechanism for recyclable, strong, and durable adhesion based on reversible assembly/disassembly of P(DPOP-Si). The key factors include the following: (i) Water-insensitive P(DPOP-Si) can be repeatedly dissolved in ethanol in a disassembly state; (ii) the self-assembly of P(DPOP-Si) can be repeatedly driven by π-π stacking during ethanol volatilization. It is well known that cross-linked poly(organosiloxane) adhesives are generally insoluble in organic solvents (*45*). However, in this study, the large steric hindrance of DPOP may limit the degree of cross-linking and polymerization of the P(DPOP-Si) adhesive, resulting in high solubility in ethanol. To confirm this conjecture, we took two poly(organosiloxanes) [named P(OTMS) and P(MTES)] with similar structures to P(DPOP-Si) and only changed the side groups for comparative study (Fig. 4A and fig. S8). Both P(OTMS) with methyl groups and P(MTES) with octyl groups show completely different dissolution behaviors from P(DPOP-Si) with DPOP groups. As the volume of the pendant groups increased, the corresponding poly(organosiloxanes) became more compatible with ethanol. As shown in Fig. 4B, in ethanol, P(OTMS) was directly precipitated, and P(MTES) was transformed into a gel state with a gel content of 73.2% and a swelling rate of 222.8% (fig. S9), while P(DPOP-Si) was dissolved with a colorless and transparent solution. In addition, the solid-state ^29^Si NMR was performed to analyze the cross-linked structure of P(DPOP-Si) (Fig. 4C). The ratio of R-Si(OSi)_3_ and R-Si(OSi)_2_(OH) was nearly 1:1, confirming that because of the large steric hindrance of DPOP, a large number of Si─OH groups do not participate in the cross-linking condensation.

On the other hand, accompanied by ethanol volatilization, π-π interactions induced by DPOP groups drive the self-assembly of P(DPOP-Si), which further contributes to the key strong cohesive energy of the adhesive. To track this assembly, we simulated its ethanol volatilization process by preparing different concentrations of P(DPOP-Si) in the range of 10 to 80%. At concentrations below 60%, all solutions exhibited a similarly clear state (fig. S10A). P(DPOP-Si) nanomicelles steadily increased from 2.0 to 8.8 nm with increasing concentration from 10 to 60%. However, when the concentration was above 60%, the nanomicelles disappeared and the solution could not flow completely (fig. S10B), indicating that large-scale assembly occurred. Similarly, the sharp change in the viscosity in the dynamic rheology also confirmed the macroscopic assembly of P(DPOP-Si) caused by ethanol volatilization (fig. S11).

To further study the π-π interactions during the assembly process, we performed ultraviolet-visible (UV/Vis) absorption characterization of P(DPOP-Si) nanomicelles at different concentrations (Fig. 4D). With higher concentrations, the intensity of the absorption peaks for DPOP groups increased notably . In particular, the obvious blueshift of the phenyl characteristic peaks evidenced the existence of π-π stacking. When the critical concentration of 60% was reached, two new sharp absorption hillocks emerged at 266 and 261 nm because of strong π-π interactions (*46*, *47*). Combined with the consistent macroscopic viscosity change, these results demonstrate that π-π stacking promotes the assembly of P(DPOP-Si) during ethanol volatilization. Note that strong π-π interactions would result in significantly different inhomogeneity of electron cloud density. Therefore, we used small-angle x-ray scattering (SAXS) tests to investigate the π-π stacking interactions of the P(DPOP-Si) adhesive (Fig. 4, E and F). Compared with P(MTES) and P(OTMS), the SAXS patterns of P(DPOP-Si) showed a much larger circle-like scattering feature and a stronger sharp scattering peak intensity, because of the presence of microphase-separated domains caused by π-π interactions (*48*, *49*). This was also confirmed by wide-angle x-ray diffraction tests. In fig. S12, there were two broad diffraction peaks centered at 13° and 21°, which were attributed to the (2, 0, 0) plane of π-π stacking and amorphous regions, respectively (*50*).

The first-principles density functional theory (DFT) calculations were carried out using the dmol3 package to study the interaction energies of different side groups: methyl, octyl, and DPOP groups in this study (*51*, *52*). Because the poly(organosiloxanes) with a cross-linked structure were complex, we optimized the molecular models as three monomers of methyltriethoxysilane (MTES), trimethoxy(octyl)silane (OTMS), and DPOP-Si (fig. S13) to analyze the interaction energies. As shown in Fig. 4G, the interaction energy of DPOP-Si (−6.65 kcal/mol) was much stronger than that of MTES (−0.07 kcal/mol) and OTMS (−2.41 kcal/mol). The bonding force induced by π-π interactions is significantly enhanced with increasing unsaturated electron content, which will drive the self-assembly behavior and promote cohesive energy. In summary, according to theoretical calculations in good agreement with experimental results, the π-π interaction driving the assembly of P(DPOP-Si) is the key to reversible strong adhesion.

### Recyclable, durable, and flame-retardant coating application

**Fig. 5.**
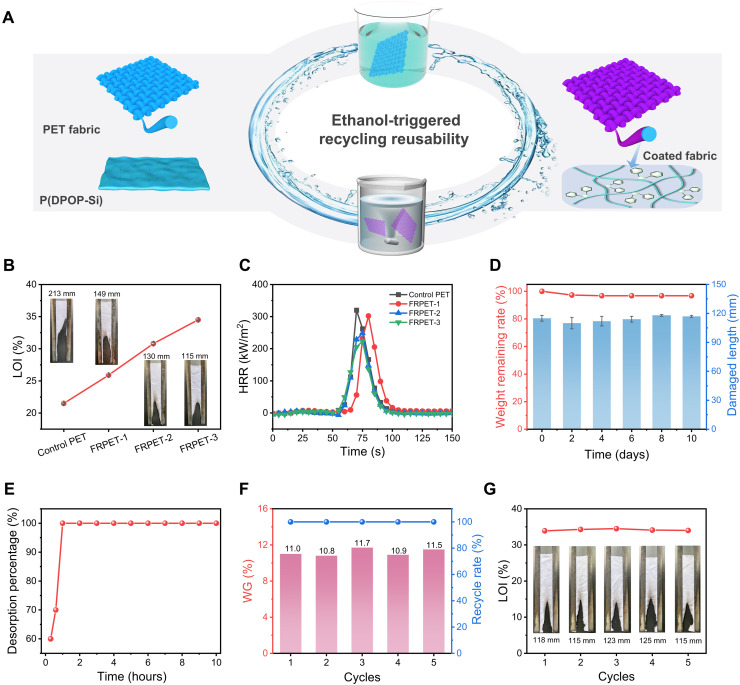
Used as protective coatings for P(DPOP-Si) adhesive. (**A**) Illustration of P(DPOP-Si) used as reversible coatings on PET fabrics. (**B**) LOI values and digital photographs after the vertical flame test for the control PET and FRPET. (**C**) Heat release rate (HRR) curves of the control PET and FRPET. (**D**) Weight remaining rates for the coating and the damaged length of FRPET after immersion in water for different days. (**E**) Desorption kinetics of P(DPOP-Si) coated on PET fabrics. (**F**) Weight gain (WG) and recycle rate of P(DPOP-Si) on PET fabrics at different reuse cycles. (**G**) LOI values and digital photographs of the recoated PET fabrics after the vertical flame tests.

Because of numerous common characterizations, adhesives can usually be used as coatings when evenly adhered to substrates on a large scale (*53*, *54*). Taking advantage of high transparency, excellent durability, recyclability, and film-forming capability, we further expand the multifunctional application of P(DPOP-Si) as a protective coating, where its flame retardancy is also evaluated (*55*). Here, the substrate is represented by PET polyester fabric, which has the largest market share in synthetic fabrics. As shown in Fig. 5A, P(DPOP-Si) can be deposited onto the surface of PET fabrics via a facile dip-coating process. After being coated, a thin P(DPOP-Si) film was wrapped around the fibers of the fabric, while the control PET fibers showed a clean and smooth surface in the scanning electron microscopy (SEM) images (fig. S14). The energy-dispersive spectroscopy (EDS) mapping and x-ray photoelectron spectroscopy (XPS) results further confirmed the highly uniform coating of P(DPOP-Si) on the PET fibers (fig. S15).

Although polyester fabrics are known to be highly flammable, a small amount of P(DPOP-Si) coating can impart durability and high flame retardancy to the fabric, which is investigated by classic limiting oxygen index (LOI), vertical flame test, and cone calorimetry test (*56*, *57*). As observed in Fig. 5B and table S3, only under the loading of 7.3 weight % (wt%) of P(DPOP-Si) coating, the LOI value of the fabric was significantly improved from the pristine 21.5 to 34.5%, showing excellent flame-retardant efficiency. The vertical flame test results (inset pictures in Fig. 5B) showed that the control PET fabrics burnt with severe dripping and flame spread hazards. In contrast, the self-extinguishing performance of the coated PET fabric was greatly improved with no droplet phenomenon and a reduced damage length (115 mm). Similarly, in the cone calorimetry test, the control PET fabrics showed a sharp heat release with a high peak heat release rate (pHRR; Fig. 5C) of 320.0 kW/m^2^ and a total heat release (THR; fig. S16 and table S4) of 5.7 MJ/m^2^. The introduction of P(DPOP-Si) coating both reduced pHRR and THR values, indicating a broad-scale flame-retardant protection function from small fire ignition to high-power thermal radiation. Combined with the increased residue and numerous pore structures after combustion (fig. S17B), the P(DPOP-Si) coating may accelerate the carbonization of the material at high temperatures to form a physical barrier and release incombustible gas to slow down the combustion process (*54*, *58*). A more detailed condense/gas dual-phase flame-retardant mechanism is shown in the Supplementary Materials (figs. S17 to S20). As the dip-coating process was usually suitable for small batches of substrates, we further evaluated the method for the large-scale preparation of coated PET fabrics, which is a key factor in practice. As observed in fig. S21A, the large-scale coated PET fabric with a size of 100 cm × 50 cm could be fabricated successfully through a facile spraying process. As expected, the spray-coated large-size PET fabric also exhibited excellent durable flame retardancy similar to those from the dip-coating process (fig. S21B and table S5), showing great potential for large-scale application.

In particular, P(DPOP-Si) exhibits excellent durability even when used as a coating. After immersion in water for 10 days (fig. S22A), the weight of the P(DPOP-Si) coating on the PET fabric almost remained unchanged (Fig. 5D). Meanwhile, XPS (fig. S23) and SEM-EDS characterization (fig. S24) indicated that the coatings maintained stable composition and morphology during soaking in water. Consequently, the P(DPOP-Si) coating shows great durable flame retardancy with water resistance, which is important for flame-retardant coatings but often difficult to achieve. Compared to the pristine fabric, the coated PET fabric after long-term soaking exhibited similar self-extinguishing behavior with no droplet phenomenon and short damage length (around 115 mm; Fig. 5D, fig. S25, and table S6). For the large-scale coated PET fabric, the P(DPOP-Si) coating retained similarly high durability. The coating showed outstanding endurance in a machine washing simulation test. Even after 50 machine washing cycles, the weight remaining rate was competitive to be as high as 97.0%, and the flame retardancy of the fabric survived perfectly from the results of the vertical flame test (fig. S25 and table S6). The durability of the P(DPOP-Si) coating showed similar performance in the abrasion resistance test (fig. S22B). Even after 50 frication cycles, the weight retention of the coating was as high as 98.7%, while the morphology, chemical structure, and flame retardancy remained unchanged (figs. S23, S26, and S27 and table S7). Overall, the P(DPOP-Si) coating endows the PET fabric with excellent flame-retardant functional durability under various harsh application conditions, which is required for functional fabrics (*59*, *60*). The strong cohesive energy from water-insensitive siloxane assemblies driven by π-π stacking ensures the strong adhesion and stability of the P(DPOP-Si) coating. Further combined with sufficient π-π interactions between the coating and PET fabric with a large number of phenyl groups, the P(DPOP-Si) coating can endow PET fabric with excellent long-term functional durability under complex conditions.

In addition to excellent durability, the closed-loop recycling of the P(DPOP-Si) coating can be easily achieved, similar to their adhesive applications. As shown in Fig. 5A, the P(DPOP-Si) coating can be removed from the fabric surface by soaking in ethanol accompanied by the disassembly process. The desorption percentage of the coating quickly reached 100% within 1 hour (Fig. 5E). The complete removal of the coating from the fabric was further confirmed by SEM-EDS mapping results (fig. S28). Subsequently, the recycling-reusing method was conducted by dipping the ethanol-washed PET fabric into P(DPOP-Si) nanomicelles according to the original coating conditions. Over the five recycling cycles, the P(DPOP-Si) coating consistently exhibited a 100% recovery rate, and each recoated fabric obtained a coating with similar weight loading (~11 wt%) and morphology (Fig. 5F and fig. S29). In addition, the recycled coating endowed the fabric with the same flame retardancy with an LOI value of 34.0 ± 0.5% and a steady damage length (120 ± 5 mm) in the vertical flame test during the reused cycles (Fig. 5G, fig. S30, and table S8). Combining high durability, transparency, recyclability, and flame retardancy (Fig. 2G), the reversibly assembled P(DPOP-Si) provides a new perspective for the sustainable development of multifunctional integrated adhesives and coatings, which will show great potential in various fields in the future.

## DISCUSSION

In summary, we demonstrate a completely closed-loop recyclable aromatic siloxane adhesive that exhibits multiple outstanding advantages including strong adhesion, excellent durability, various harsh-condition resistance, transparency, and flame retardancy. Driven by the π-π stacking of aromatic side groups, the designed P(DPOP-Si) adhesive can be assembled into an ordered layered micromorphology on the bonding surface, resulting in high transparency (98%), low haze (2.5%), and strong cohesive energy. Consequently, the P(DPOP-Si) adhesive exhibits strong and durable adhesion (up to 3.5 MPa) on various materials. Notably, the strong adhesion can be long-term maintained without decay even in harsh conditions of high humidity (85% RH), in underwater, salt, and acid/alkali solutions (pH 1 to 14), and at low/high temperatures (−10° to 90°C). By applying infiltration-volatilization of ethanol, the adhesives can be completely closed-loop recycled with a 100% recovery rate and reused more than 100 times, where the repeated assembly/disassembly of P(DPOP-Si) occurs. In addition, because of the presence of phosphorus, P(DPOP-Si) can be directly used as a flame-retardant protective coating, imparting self-extinguishing properties to the polyester fabric while maintaining high transparency, durability, and full recyclability. The corresponding mechanism analysis based on experimental results and theoretical simulation calculations confirms that the water-insensitive π-π stacking interaction driving the reversible assembly enables closed-loop recycling and durable strong adhesion of P(DPOP-Si). This study not only offers a π-π stacking–driven siloxane assembly strategy to achieve reversible, strong, and durable adhesion in harsh conditions but also provides new insight into the sustainable development of adhesives and coatings with circular life multifunctional integration.

## MATERIALS AND METHODS

### Materials

Polyester fabrics (100% PET; 86 g/cm^2^) were purchased from the Aotai Textile Sales Company, China. DPOP and VTES were obtained from Shanghai Aladdin Biochemical Technology Co. Ltd., China. MTES and OTMS were supplied by Shanghai Aladdin Biochemical Technology Co. Ltd., China. Sodium hydroxide (NaOH) was obtained from Tianjin Beichenfangzheng Chemical Co. Ltd., China. Ethanol (AR), butanol, hexanol, acetone, THF, DMSO, TCM, toluene, *n*-hexane, and PE were supplied by Chengdu Kelong Chemical Co. Ltd., China. All materials were used without further treatment.

### Synthesis of DPOP-Si and P(DPOP-Si) adhesive

DPOP-Si was synthesized via the addition reaction between DPOP and VTES in a 500-ml three-necked flask equipped with a condenser, a mechanical stirrer, and a thermometer. In detail, DPOP (20.2 g, 0.10 mol) and VTES (19.3 g, 0.10 mol) were introduced into a flask containing 20 ml of TCM. The reaction mixture was heated to 80°C and stirred for 18 hours under an N_2_ atmosphere. After removing the solvent by rotary evaporation, DPOP-Si was obtained with overall yields of >90%.

The P(DPOP-Si) adhesive was prepared by the hydrolysis-condensation reaction of DPOP-Si. Briefly, DPOP-Si was dissolved into a mixture medium of ethanol/water (v/v; 2:1) with a concentration of 30% and constantly stirred at 80°C for 8 hours until a homogeneous and transparent P(DPOP-Si) precursor was obtained. Then, most of the solvent was evaporated to achieve the P(DPOP-Si) adhesive for further adhesion application and study.

### Preparation of adhesion samples and lap shear tests

According to the standard ASTM F2255, the P(DPOP-Si) adhesive was first cast on the surface of one substrate with an area of 1 cm × 2 cm. Subsequently, another substrate was pressed on the adhesive area at a force of approximately 2 N until the adhesive set. After complete drying, the sample for the adhesion strength measurement was obtained. The lap shear tests were conducted using a universal testing machine at a strain rate of 5 mm/min.

### Recycling-reusing methods for P(DPOP-Si) adhesives on wood surfaces

A quantity of the P(DPOP-Si) adhesive was first adhered to the wood substrate. When recovered, the adhesive was treated and dissolved with excess ethanol to remove it from the wood surface, resulting in a homogeneous solution. Then, the ethanol was evaporated to achieve the recycled P(DPOP-Si) adhesive for the next adhesion application.

### Swelling tests

A certain quality of the P(MTES) gel sample was taken and swelled in ethanol for 24 hours, followed by extraction at room temperature, and dried at 80°C. Then, the masses of the original samples (*m*_0_), the swelled extracted samples (*m*_s_), and the dried extracted samples (*m*_d_) were recorded. The swelling ratio [*S* (%)] and gel content [*G* (%)] were calculated by the following formulas

$$S(\%)=\frac{m_{s}}{m_{d}}\times 100\%$$

$$G(\%)=\frac{m_{d}}{m_{0}}\times 100\%$$

### DFT calculations

The first-principles DFT calculations were carried out using the dmol3 package. The electron exchange function was calculated using Perdew-Burke-Ernzerhof described by the generalized gradient approximation. An all-electron double numerical atomic orbital augmented by double numerical plus polarization *n* was used as the basis set, and DFT semicore pseudo-potentials were used for the interactions between the ion cores. The convergence criteria in total energy, maximum force, and maximum displacement were set at 10^−5^ Hartree, 0.002 Hartree/Å, and 0.005 Å, respectively. The electronic self-consistent field tolerance was set at 10^−6^ Hartree.

Accordingly, the interaction energy is defined as follows

$$E_{\mathrm{ads}}=E\;\left(\mathrm{total}\right)-E\left(a\right)-E(b)$$

where *E*_ads_ is the adsorption energy of DPOP-Si, MTES, and VTES; *E* (total) is the energy of the molecule and itself (DPOP-DPOP, CH_3_-CH_3_, and octyl-octyl); and *E* (a) and *E* (b) are the single-point energies of the molecule.

### Fabrication of flame-retardant polyester (PET) fabrics coated with P(DPOP-Si)

The polyester (PET) fabrics, named control PET, were pretreated as follows: The PET fabrics were soaked in NaOH solution (5 wt%) at 60°C for 1 hour. After rinsing with deionized water and drying at 80°C for 24 hours, the control PET (pretreated PET fabrics) was obtained.

Then, the control PET fabric was dipped into a certain concentration P(DPOP-Si) precursor with a material-to-liquid ratio of 1:20 at 60°C for 30 min and dried at 80°C for further hydrolysis-condensation reactions on the fabric surface until a constant weight was achieved. The weight gain (WG) was calculated by the following formula: WG (%) = (*w*_1_ − *w*_0_)/*w*_0_ × 100%, where *w*_0_ represents the weight of the control PET fabrics and *w*_1_ represents the weight of the coated fabrics. Here, the coated PET fabrics with WG of 5.0, 6.0, and 7.3% were obtained by adjusting the concentration of the P(DPOP-Si) precursor to 5.0, 7.5, and 10.0%, which were named FRPET-1, FRPET-2, and FRPET-3, respectively. The WG of the fabric exhibits a high linear correlation with increasing P(DPOP-Si) concentration (fig. S31 and table S1).

The spraying coating process is used to fabricate large-scale coated PET fabrics. A 10% concentration of P(DPOP-Si) in ethanol was transferred into a spray bottle. Subsequently, the double-sized spraying was conducted from a height of 15 to 20 cm above the PET fabric with a size of 100 cm × 50 cm. The resulting fabric was further dried at 80°C. By adjusting the spray time and program, a coating weight loading of about 11 wt% was achieved. The large-scale coated PET fabric was named LFRPET.

### Desorption of P(DPOP-Si) coatings from PET fabrics

To investigate the desorption behaviors, the coated PET fabrics (FRPET-3) with a fixed WG of ~7 wt% were soaked in excess ethanol for different times and then extracted, followed by drying at 80°C until achieving a constant weight. The desorption was characterized by measuring the percentage of P(DPOP-Si) coatings removed from the PET fabrics.

### Durability tests

To assess the durability against water, the coated PET fabrics (FRPET-3) and LFRPET were soaked in water for several days. After that, the fabrics were extracted and dried at 80°C for 2 hours for further study. In addition, the fabrics soaked in water were named FRPET-3-W and LFRPET-W, respectively.

For the machine washing simulation test, the LFRPET sample was stirred vigorously in excessive water at 45° ± 2°C at a stirring speed of 1000 rpm and dried, which was taken as one cycle. Then, the samples were processed repeatedly for 50 cycles (total machine wash time of 25 hours). The LFRPET sample after washing was named LFRPET-MW.

An abrasion resistance test was also used to investigate the durability of the P(DPOP-Si) coatings. As shown in fig. S22B, a piece of sandpaper was placed on the coated PET fabrics (FRPET-3) with a load of 500 g and moved over the entire length along the lengthwise direction of the fabrics. A back and forth was recorded as one cycle of abrasion. After 50 cycles, the fabrics were turned over, and another 50 cycles of abrasion were completed. The sample after the abrasion resistance test was named FRPET-3-A.

### General characterization

FTIR spectra were obtained on a Nicolet 6700 instrument (Thermo Fisher Scientific Co., USA) with wave numbers ranging from 400 to 4000 cm^−1^. ^1^H NMR spectra were obtained on a Bruker AV II 400-MHz spectrometer (Bruker, Switzerland) using DMSO as the solvent. Solid-state ^29^Si NMR investigation was carried out on a Bruker-600 NMR 600-MHz spectrometer (Avance, Bruker, Switzerland). The degree of condensation (DC) was calculated from the peak areas of *T*^1^ [R-Si(OSi)(OH)_2_], *T*^2^ [R-Si(OSi)^2^(OH)], and *T*^3^ [R-Si(OSi)_3_] based on the following equation: DC = (*T*^1^ + 2*T*^2^ + 3*T*^3^)/3.

SEM was performed on JSM-7500F (JEOL) at an accelerating voltage of 15 kV. SEM was coupled with an energy-dispersive x-ray spectrometer (EDX) to analyze element compositions and distribution maps. Before the test, the surfaces of the samples were first coated with a thin layer of gold. TEM images were captured with JEM-HT7700 (JEOL). UV-Vis spectra of the samples were obtained on a UV1800 UV-Vis scanning spectrophotometer (Shimadzu, Japan). Micelle size was measured with Zetasizer Nano ZS (Malvern). Wide-angle x-ray scattering was examined with a Philips X’Pert Pro diffractometer at room temperature. The SAXS experiments were recorded on a Xeuss 2.0 diffractometer. The shear adhesion strength was investigated by using a SANS universal testing machine (CMT 6000). The LOI test was performed on the HC-2C oxygen index instrument according to the standard method DIN EN ISO 15025, and the specimen dimensions were 160 mm × 100 mm. A vertical flammability test was carried out with specimen dimensions of 300 mm × 80 mm on a CZF-3 instrument (Jiangning, China) according to the GB/T 5455-1997 standard method. The cone calorimeter test was performed with a cone calorimeter at a heat flux of 35 kW/m^2^ according to the standard method ISO 5660-1. The sample used for the test was 100 mm × 100 mm × 0.2 mm. Rheological measurements were performed on Discovery HR-2 (TA Instruments, USA) at a fixed temperature of 25°C. Thermogravimetry–infrared spectrometry was performed with a TA TGA5500 thermogravimetric analyzer that was linked to a Nicolet iS50 FTIR spectrophotometer at a heating rate of 20°C/min from 40° to 700°C in a nitrogen atmosphere (flow rate: 25 ml/min). Raman spectra were observed with a laser Raman spectrometer (LabRAM HR, HORIBA Co., France) in the range of 800 to 2000 cm^−1^ with an excitation wavelength of 532.17 nm and an integration time of 10 s. XPS was measured on a XSAM 800 spectrometer (KRATOS, UK) using Al Ka excitation radiation (1486.6 eV).

## Supplementary Materials

### This PDF file includes:

Supplementary Text Figs. S1 to S31 Tables S1 to S8 References
