# Supplementary material for: Fully recyclable multifunctional adhesive with high durability, transparency, flame retardancy, and harsh-environment resistance
Source: Sci Adv. 2022 Dec 14;8(50):eadd8527. doi: 10.1126/sciadv.add8527 (PMC9750157; doi:10.1126/sciadv.add8527)
Supplement: Supplementary file 1 — Supplementary Text Figs. S1 to S31 Tables S1 to S8 References [file sciadv.add8527_sm.pdf]

Science Advances
AAAS

Supplementary Materials for

Fully recyclable multifunctional adhesive with high durability, transparency, flame retardancy, and harsh-environment resistance

Zi-Hao Wang *et al.*

Corresponding author: Hai-Bo Zhao, haibor7@163.com; Xiu-Li Wang, xiuliwang1@163.com

Sci. Adv. **8**, eadd8527 (2022)
DOI: 10.1126/sciadv.add8527

The PDF file includes:

Supplementary Text
Figs. S1 to S31
Tables S1 to S8
Legends for movies S1 to S3
References

Other Supplementary Material for this manuscript includes the following:

Movies S1 to S3

Supplementary Text

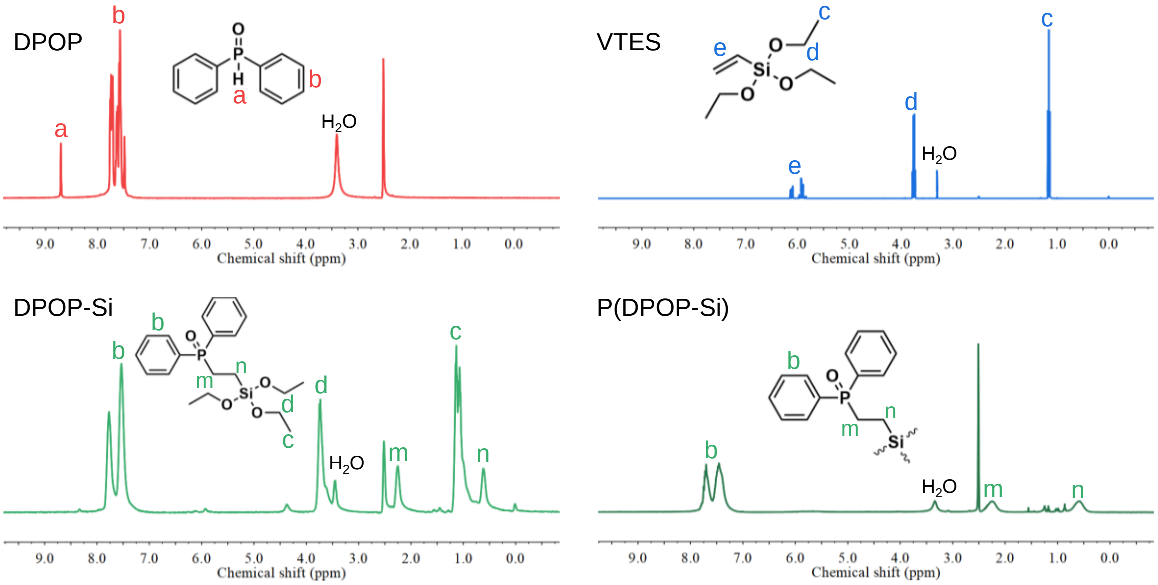

Fig. S1. ^{1}H NMR characterization. ^{1}H NMR spectra of reactants, DPOP-Si, and P(DPOP-Si).

The chemical structures of DPOP-Si and P(DPOP-Si) were characterized via ^{1}H NMR spectroscopy (**Fig. S1**). For the DPOP-Si spectra, the characteristic signals of P-H (8.47 ppm, peak a) and C=C (5.90-6.21 ppm, peak e) attributed to DPOP and VTES, respectively, disappeared. Instead, there were two new signals at 2.30 and 0.85 ppm (peaks m and n), which corresponded to the methylene protons of Si-CH_2 and P-CH_2. In addition, the -CH_2CH_3 group signal (peaks d and c) almost disappeared, indicating that the hydrolysis-condensation reaction was carried out successfully.

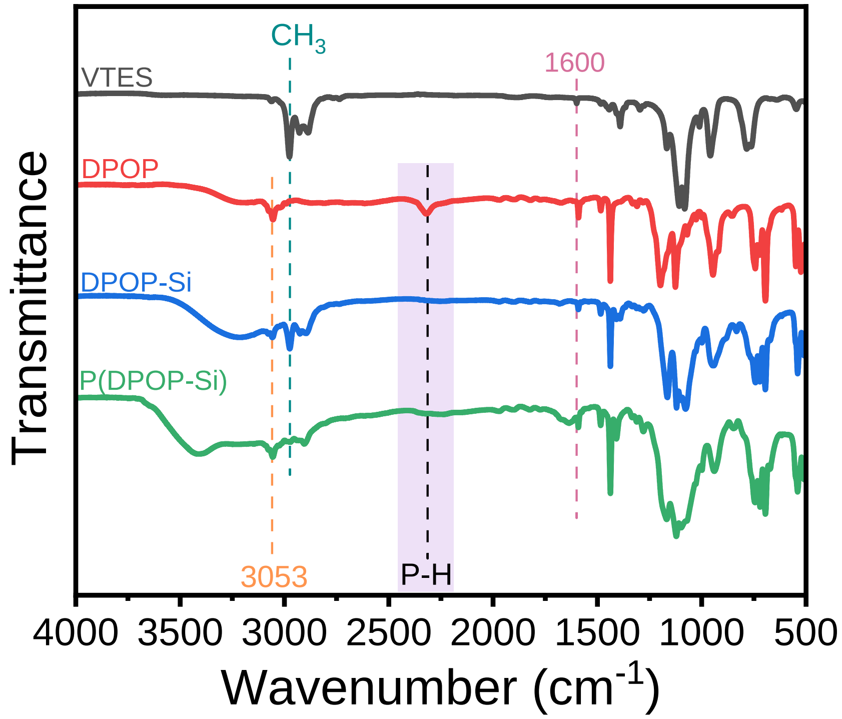

Fig. S2. FT-IR characterization. FT-IR spectra of reactants, DPOP-Si, and P(DPOP-Si).

The FTIR spectrum (**Fig. S2**) also showed that the two characteristic peaks of P-H and C=C at 2314 and 1600 cm^{-1} disappeared in the spectrum of DPOP-Si. In addition, the characteristic signal at 2977 cm^{-1} attributed to $-CH_3$ also disappeared after the hydrolysis-condensation reaction. All these results clearly revealed that DPOP-Si and P(DPOP-Si) were prepared successfully.

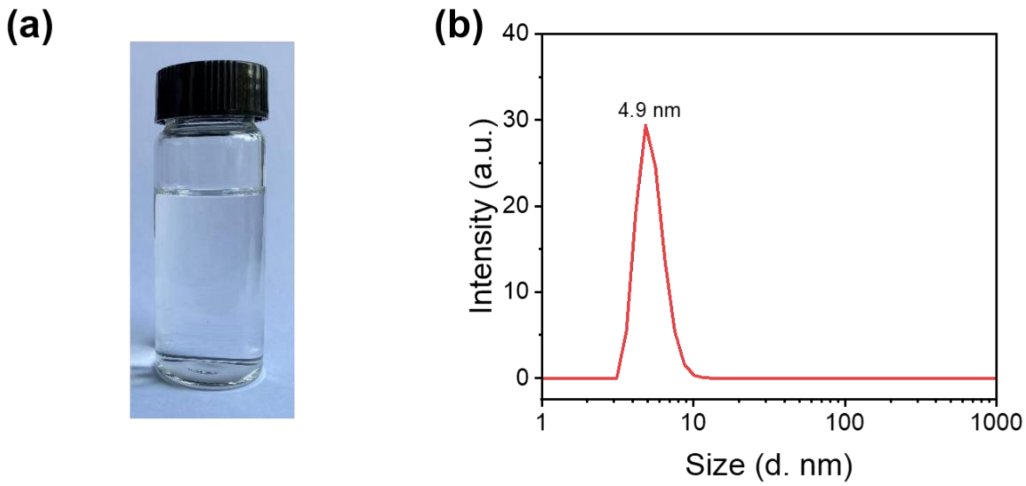

Fig. S3. Dissolution behavior for P(DPOP-Si) in ethanol. (a) Digital photos of P(DPOP-Si) precursor. (b) Particle size of nanomicelles in P(DPOP-Si) precursor.

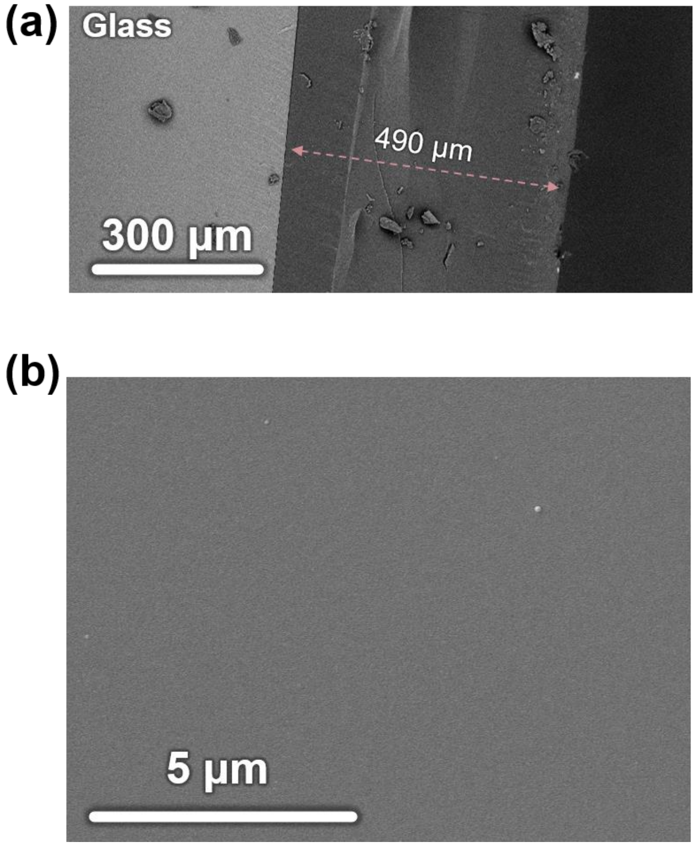

Fig. S4. Microstructure of P(DPOP-Si) on the glasses. SEM images for the (a) cross-section and (b) top surface of P(DPOP-Si) adhesive.

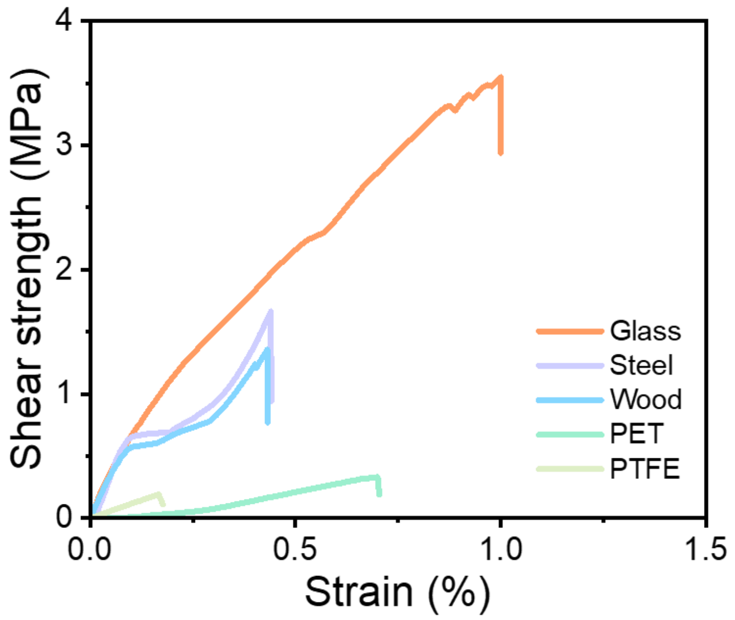

Fig. S5. Adhesion properties for different substrates. Lap shear stress–strain curves of P(DPOP-Si) adhesive on various substrate surfaces (glass, steel, wood, PET, and PTFE).

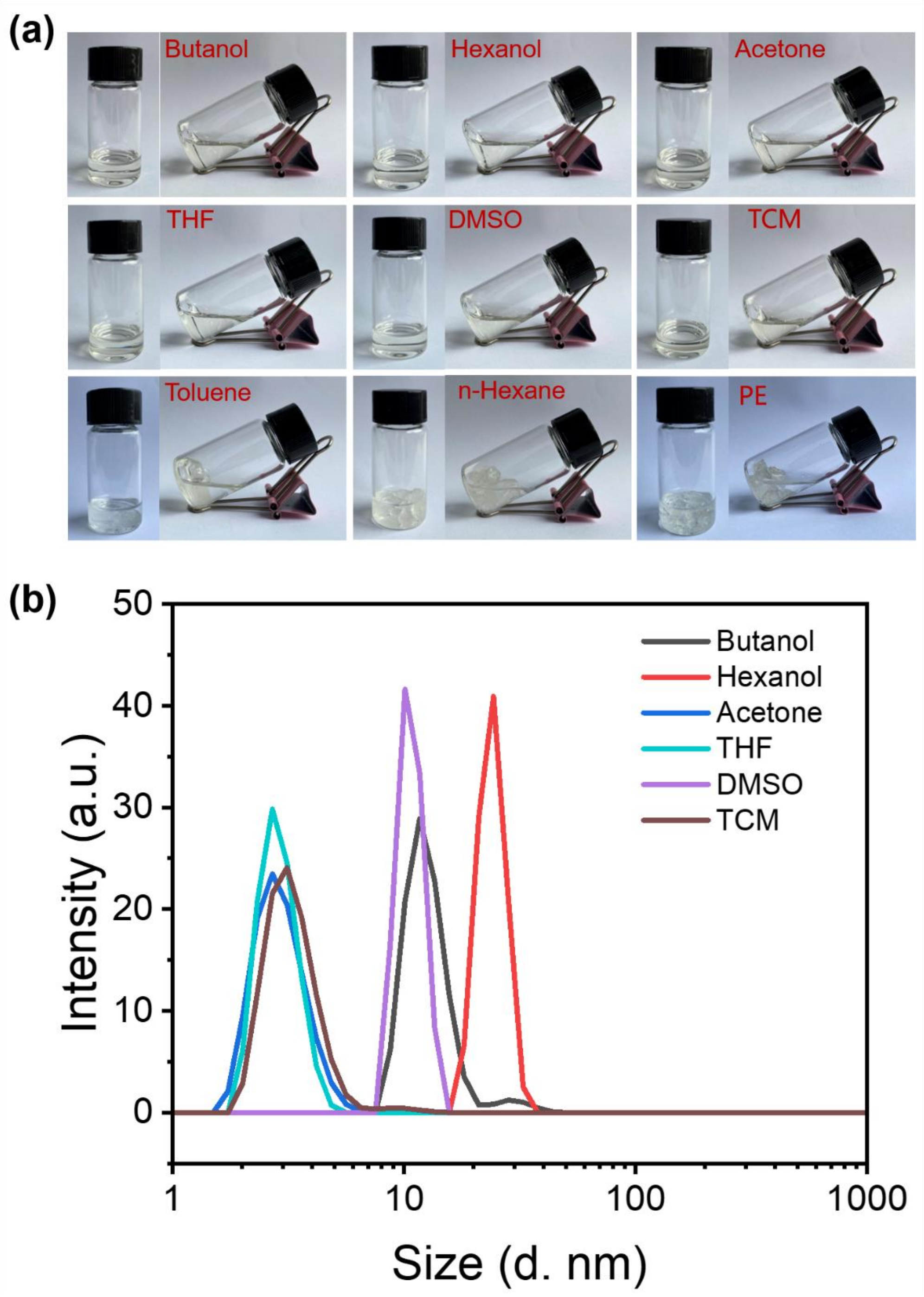

Fig. S6. Dissolution behavior for P(DPOP-Si) in different typical organic solvents. (a) Digital photographs of the dissolution behavior for P(DPOP-Si) in different typical organic solvents. (b) Particle sizes of P(DPOP-Si) nanomicelles in different organic solvents.

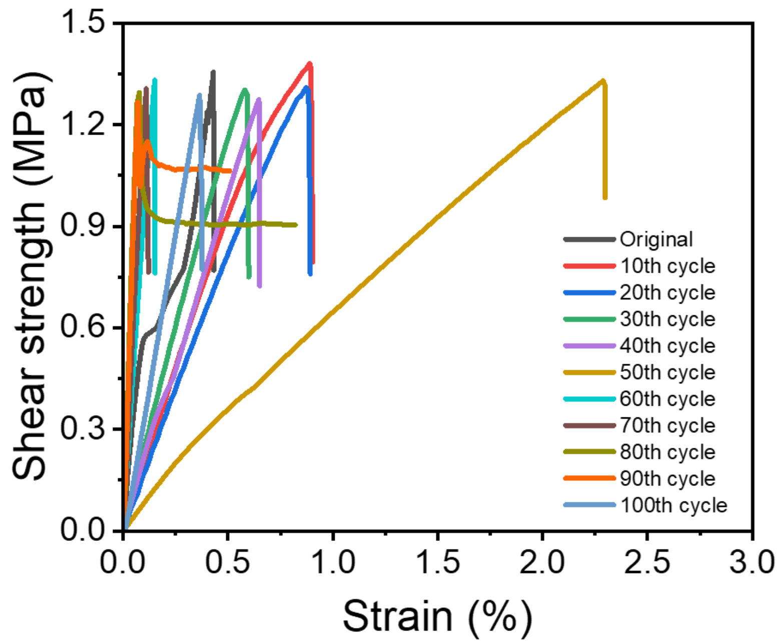

Fig. S7. Adhesion properties of the recycled P(DPOP-Si) adhesive. Lap shear stress–strain curves of the reused adhesion test for P(DPOP-Si) adhesive on the wood surface.

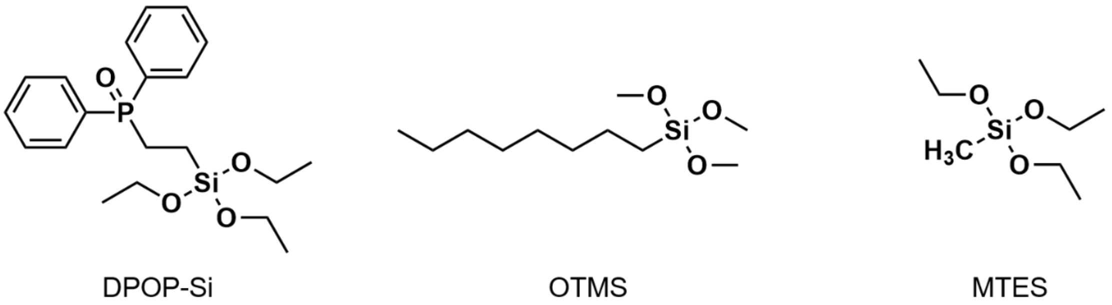

Fig. S8. Different molecular structures of silicon precursors. Molecular structures of DPOP-Si, OTMS, and MTES.

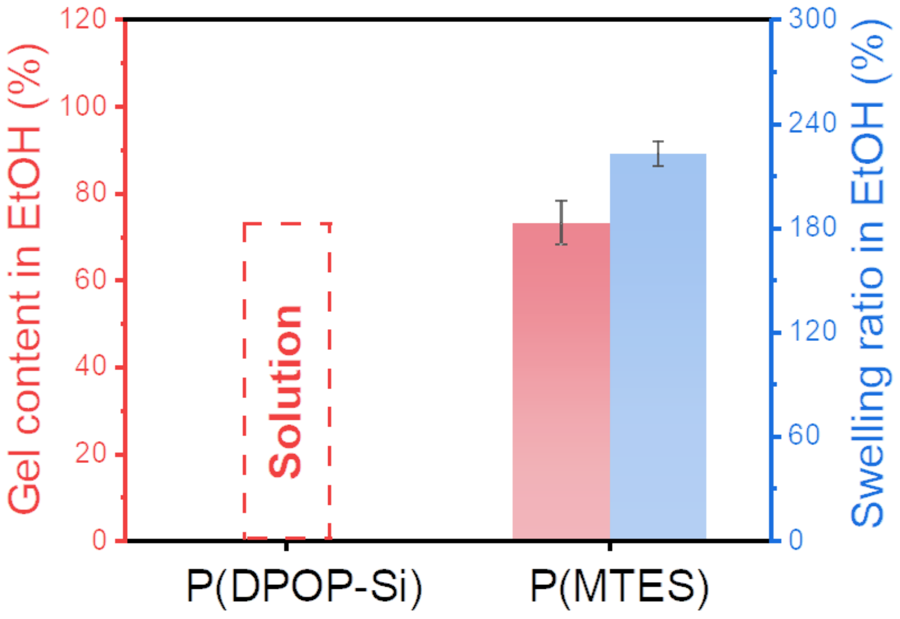

Fig. S9. Swelling performance for P(MTES) in ethanol. Gel content and swelling ratio of P(MTES) in ethanol.

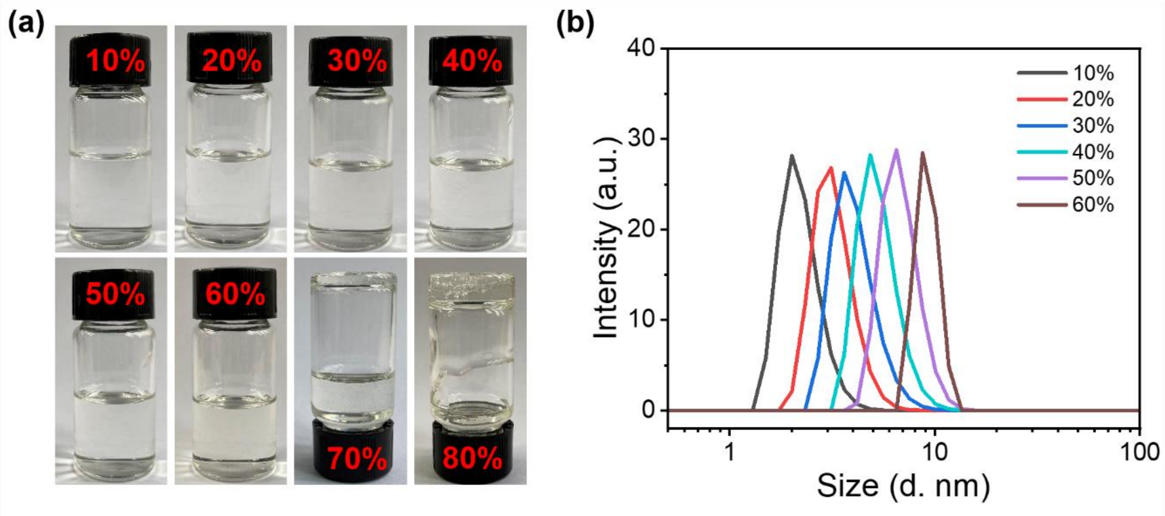

Fig. S10. Dissolution behavior for different concentrations of P(DPOP-Si)/ethanol solutions. (a) Digital photos of different concentrations of P(DPOP-Si)/ethanol solutions. (b) Particle sizes of nanomicelles for different concentrations of P(DPOP-Si)/ethanol solutions.

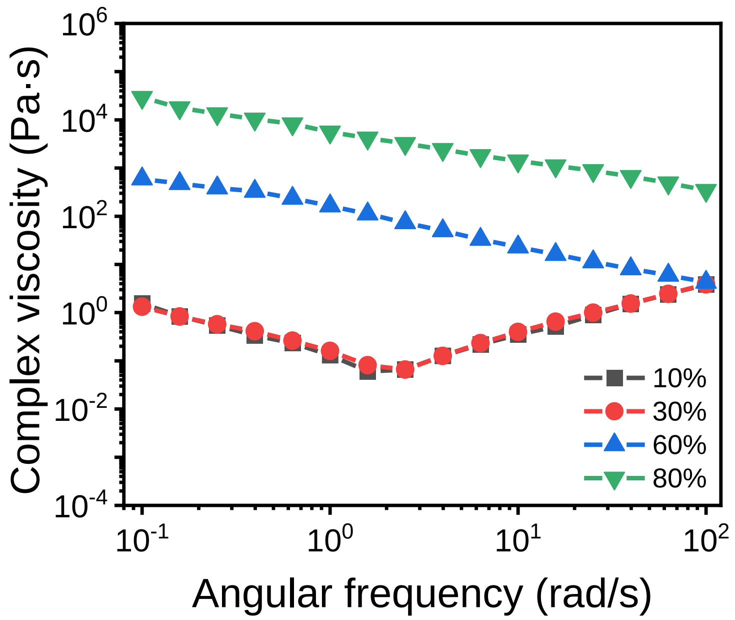

Fig. S11. Rheological behavior for different concentrations of P(DPOP-Si)/ethanol solutions. Frequency dependence of complex viscosity for P(DPOP-Si)/ethanol solutions with different concentrations (10%, 30%, 60%, and 80%).

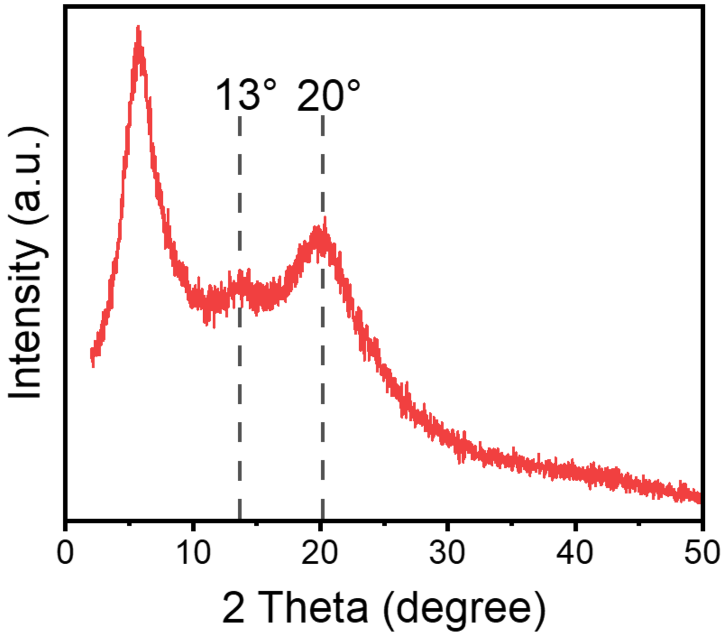

Fig. S12. WAXD pattern of P(DPOP-Si) adhesive.

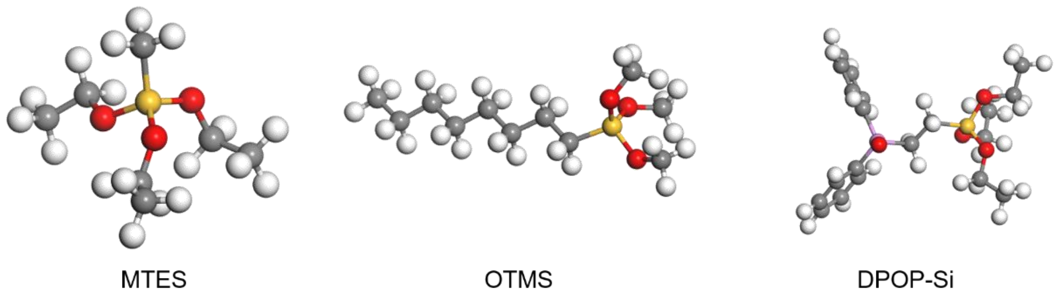

Fig. S13. DFT-optimized molecular models. (a) MTES, (b) OTMS, and (c) DPOP-Si.

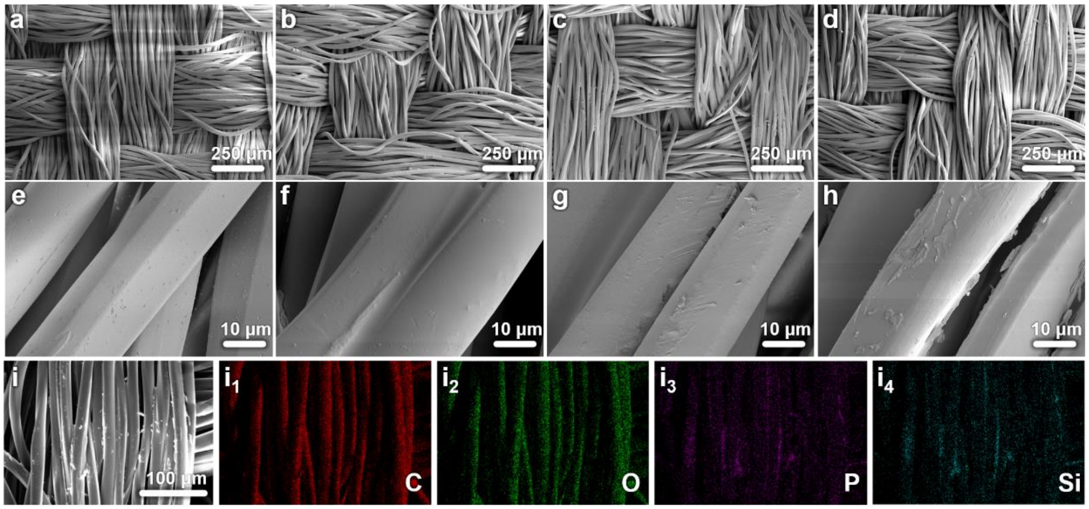

Fig. S14. Surface microstructure of control and coated PET. SEM micrographs of (a, e) control PET, (b, f) FRPET-1, (c, g) FRPET-2, (d, h) FRPET-3, and (i) C, O, P and Si element distribution maps of FRPET-3.

SEM was utilized to investigate the micromorphology of control and coated PET fabrics. As shown in **Fig. S14**, control PET fabrics displayed a clean and smooth surface morphology with some obvious gaps between the fibers. After being coated, it could still hold a complete and clear woven structure, as seen from the low-magnification SEM images. At the high magnification, it was seen that some thin and folded films tightly wrapped on the fiber surface and filled the gaps. In addition, EDS mapping results showed that N, P, and Si elements were distributed uniformly on the surface, indicating that P(DPOP-Si) adhesive could be evenly deposited on the surface of PET fabrics as a protective coating.

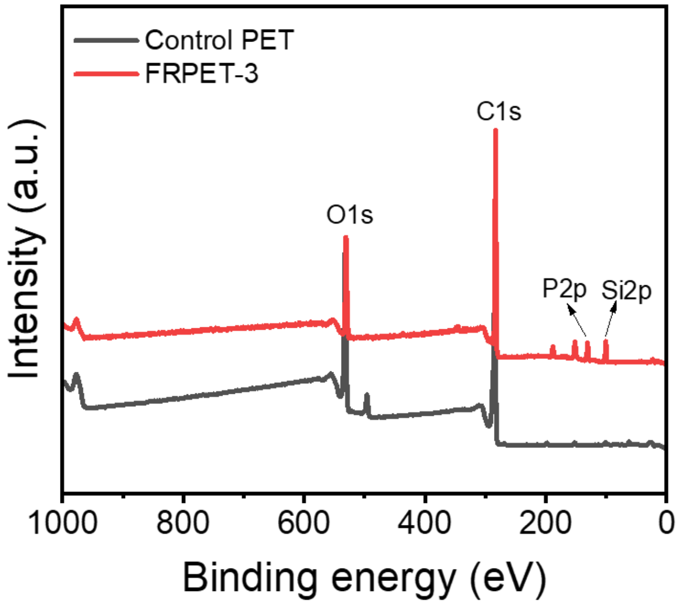

Fig. S15. XPS characterization of control and coated PET. Full survey XPS spectra of control PET and FRPET-3.

XPS was also used to prove the existence of the P(DPOP-Si) coating (**Fig. S15**). Compared with the control PET, the newly appeared P and Si elements in P(DPOP-Si) were confirmed by peaks at 130.8 eV (P2p) and 100.3 eV (Si2p), respectively. This result indicated the successful deposition of the P(DPOP-Si) coating on the surface of PET fabrics.

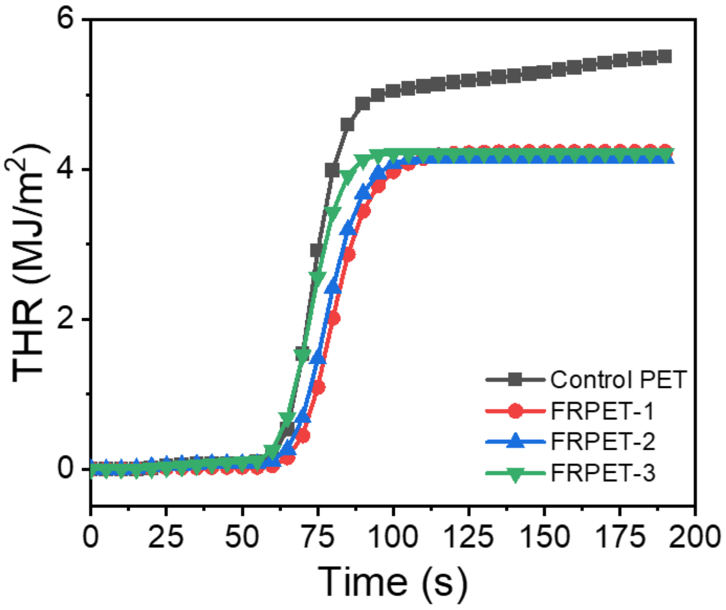

Fig. S16. THR performance for control and coated PET. THR curves for control PET and FRPET.

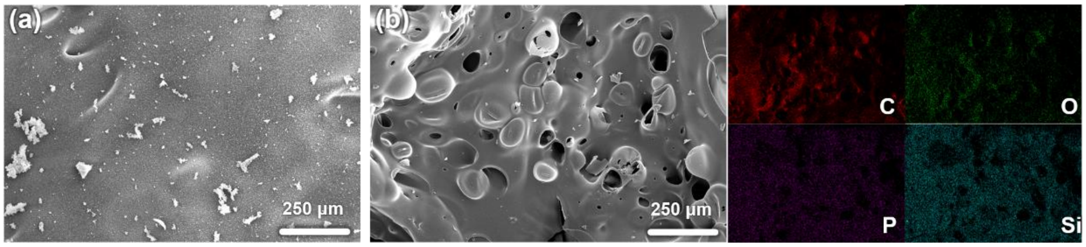

Fig. S17. Surface microstructure for control and coated PET after cone calorimeter testing. (a) SEM micrographs for the control PET after cone calorimeter testing. (b) SEM micrographs and element distribution maps for FRPET-3 after the cone calorimeter test.

The residual char after the cone calorimeter test was collected to understand the condensed flame-retardant mechanism by SEM and EDS mapping spectroscopy (**Fig. S17**). From **Fig. S17a**, it was speculated that the control PET fibers first melted and fused together during the burning process, finally causing a smooth and compacted surface. However, in sharp contrast, the coated PET fibers melted with some bubbles and pores at the micron scale appearing on the surface. This phenomenon was conducive to the release and diffusion of many nonflammable and volatile components from the P(DPOP-Si) coating, which was expected to cut across the molten zone during the burning stage. In addition, EDS mapping results exhibited well-distributed P and Si elements in the char layer, reflecting the classic condense-dominated action.

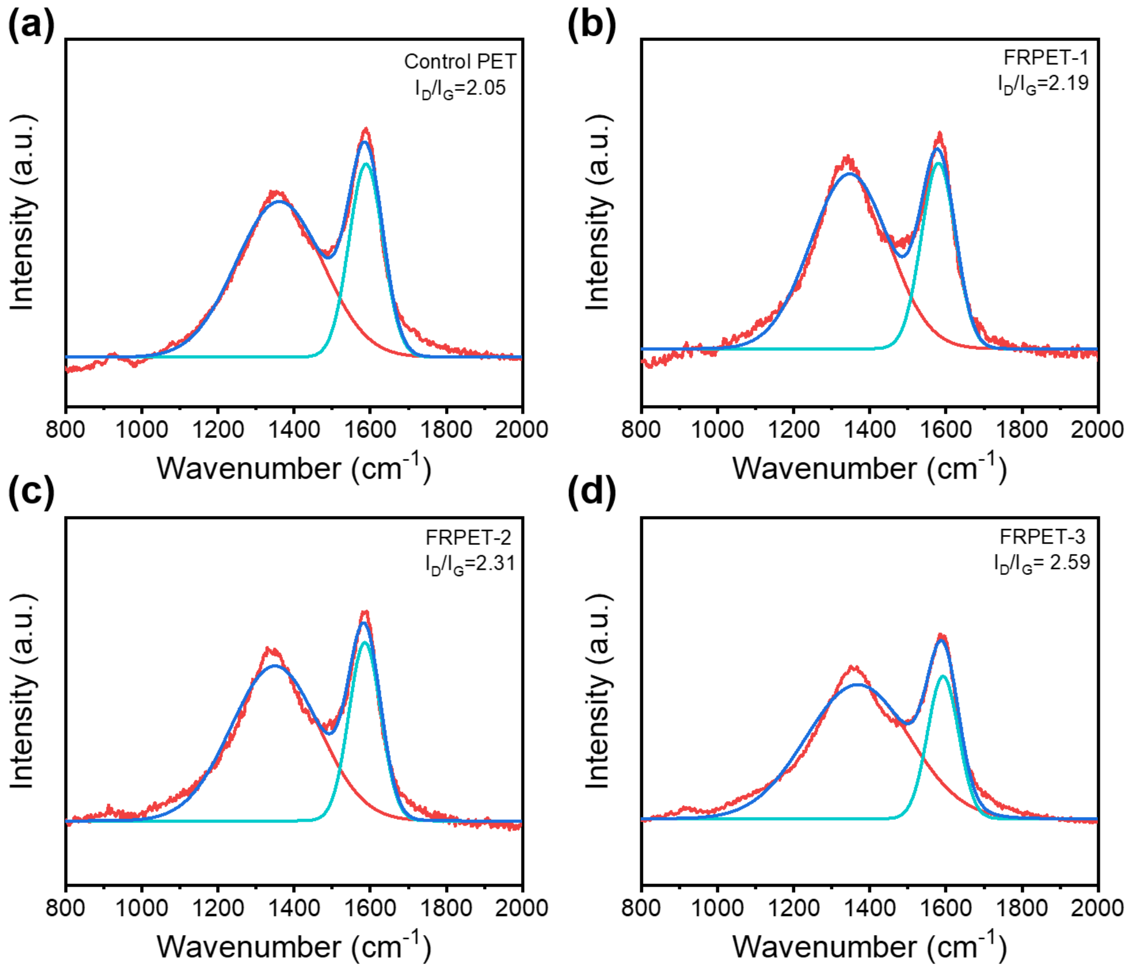

Fig. S18. Raman curves. (a) control PET and (b-d) FRPET after the cone calorimeter test.

Raman spectroscopy of all samples was utilized to analyze the char structure. There were two important peaks of carbon at 1360 cm^{-1} and 1580 cm^{-1} which were assigned to the D and G band absorption peaks, respectively. The D band represents the defective graphitized carbon, while the G band was assigned to the SP_2 hybridized carbon structure *(61)*. The larger intensity ratio of the D and G bands (I_D/I_G) reveals that a smaller crystallite and denser carbon layer was formed *(62, 63)*. In **Fig. S18**, the I_D/I_G value clearly increased with higher P(DPOP-Si) loading. FRPET-3 showed the largest I_D/I_G value of 2.59, which was much higher than that of the control PET (2.05). The result indicated that the P(DPOP-Si) coating would be more helpful for PET to form a smaller crystalline structure and a denser carbon layer during the burning process.

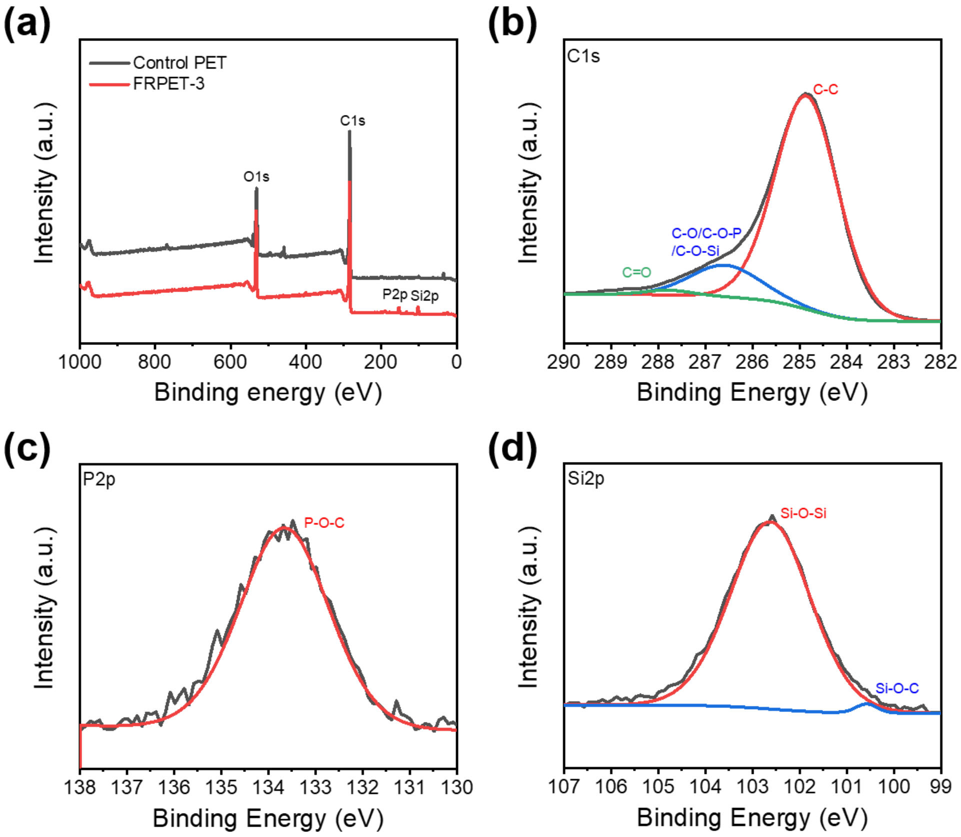

Fig. S19. XPS characterization of coated PET after cone calorimeter testing. (a) Full survey XPS and high-resolution spectra of (b) C1s, (c) P2p, and (d) Si2p of FRPET-3 after the cone calorimeter test.

The chemical composition and structure of the residual char after the cone calorimeter test were also identified by XPS characterization. The full survey spectrum, C1s, P2p, and Si2p spectrum are summarized in **Fig. S19**. **Fig. S19a** reveals that P and Si elements in the P(DPOP-Si) coating are retained in the residue char after the burning process. The C1s spectrum was divided into three peaks at 284.8 eV, 286.7 eV and 288.0 eV, which can be assigned to C-C, C-O/C-O-P/C-O-Si and C=O linkages *(64)*. The peak at 133.7 eV in the P2p spectrum was assigned to the P-O-C linkage, indicating the carbonization ability of the P(DPOP-Si) coating during combustion. In addition, the Si2p spectrum was expected to be divided into two peaks at 102.7 eV and 100.6 eV, which can be attributed to Si-O-Si and Si-O-C groups *(65, 66)*. Combining all these results, it was clear that P(DPOP-Si) can promote the formation of more protective phosphate- and silica-containing hybrid char layers during combustion, showing a condensed flame-retardant mechanism.

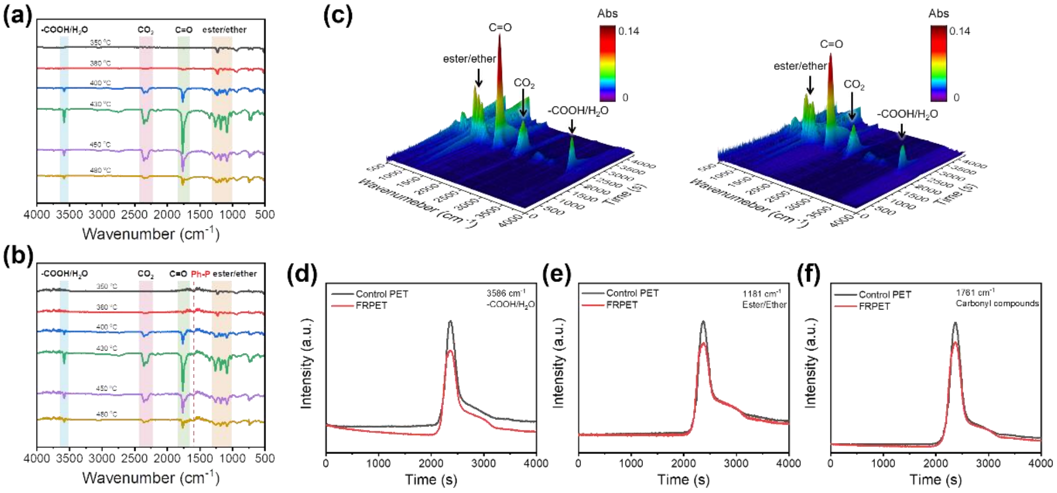

Fig. S20. Gas flame-retardant mechanism. FT-IR spectra of the released gases for (a) control PET and (b) FRPET-3 at selected temperatures; (c) 3D diagram of the volatiles in the thermal degradation of control PET and FRPET-3; (d-f) absorption intensities of the selected volatiles in the thermal degradation process of PET fabrics.

To further investigate the gas phase flame-retardant mechanism, a TG-IR test was conducted to analyse the evolved gas products of the control coated PET fabrics during the whole thermal degradation process. **Fig. S20** presents the 3D FT-IR and several corresponding FT-IR spectra of the released volatiles at selected temperatures. The control and coated PET fabrics presented the similar characteristic signals, which can be attributed to -COOH/H_2O (3580 cm^{-1}), CO_2 (2337 cm^{-1}), carbonyl compounds (1763 cm^{-1}), and ester/ether (1014-1301 cm^{-1}), respectively. However, compared with the control PET, the coated PET fabrics showed a lower characteristic absorption intensity of flammable volatile products, such as -COOH, ester/ether, and carbonyl compounds. Furthermore, the new Ph-P segment absorption peak was also detected at 1594 cm^{-1}, which may act in the gas phase flame-retardant mechanism through the free radical capture effect *(64, 67)*. Therefore, the P(DPOP-Si) coating can decrease the release of flammable volatiles and produce phosphorous-containing groups to suppress the fuel supply for burning, exhibiting efficient gas-phase flame retardancy.

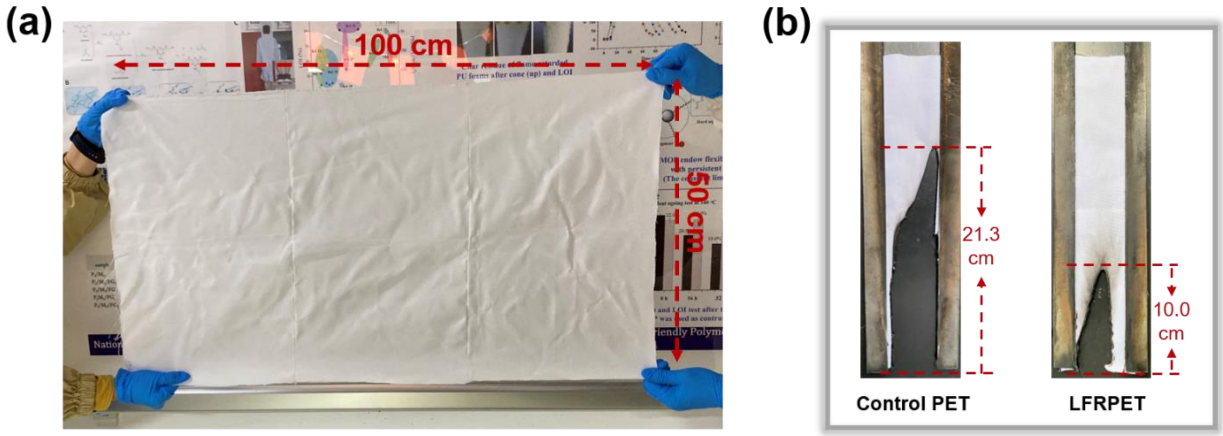

Fig. S21. Large-scale fabrication for coated PET. (a) Digital photographs of the large-scale coated PET fabric with a size of 100 cm × 50 cm (LFRPET). (b) Digital photographs after the vertical flame tests for control PET and LRPET.

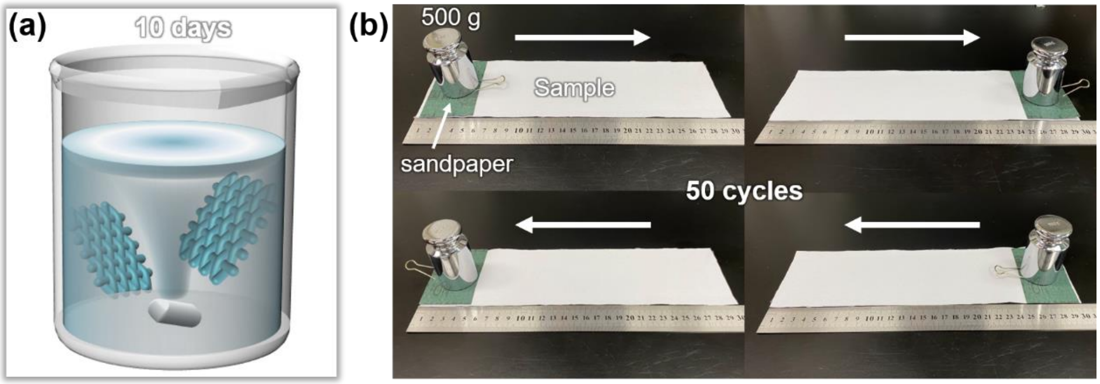

Fig. S22. Durability tests of coated PET. Implementation method of (a) immersion-in-water test and (b) abrasion resistance test.

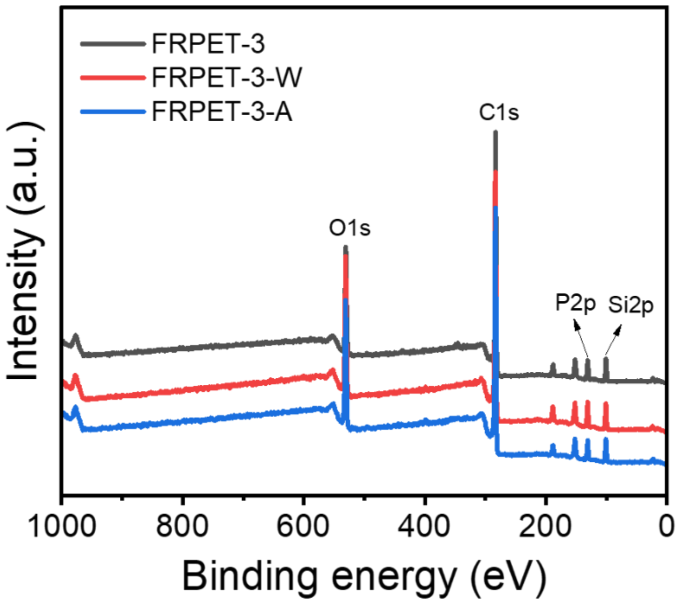

Fig. S23. XPS characterization for coated PET after durability test. Full survey XPS spectra for FRPET-3 and FRPET-3 after immersion in water (FRPET-3-W) and the abrasion resistance test (FRPET-3-A).

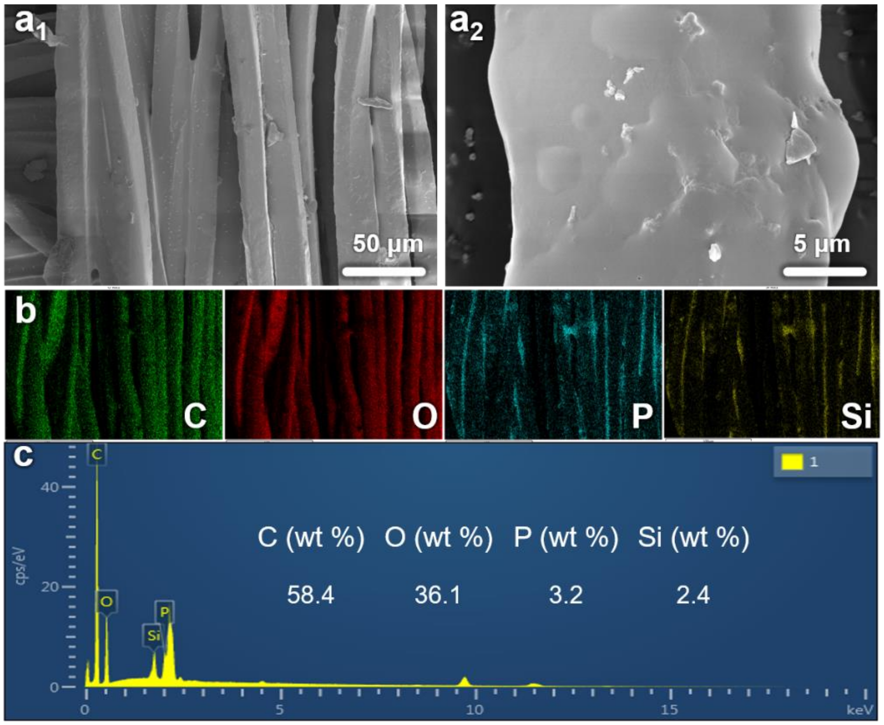

Fig. S24. Surface microstructure for coated PET after immersion in water. (a_1, a_2) SEM micrographs and (b) C, O, P and Si element distribution maps for FRPET-3 after the immersion-in-water test. (c) EDS results and element contents for FRPET-3 after the immersion-in-water test.

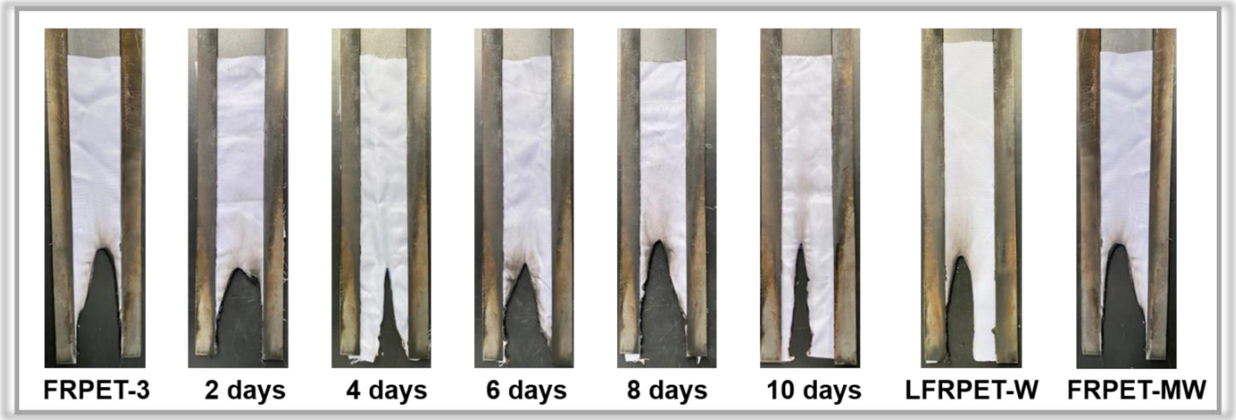

Fig. S25. Flame retardancy for coated PET after immersion in water and simulation machine washing. Digital photographs after the vertical flame tests for FRPET-3 after immersion in water for different days, LFRPET after immersion in water for 10 days and simulation machine washing for 50 cycles.

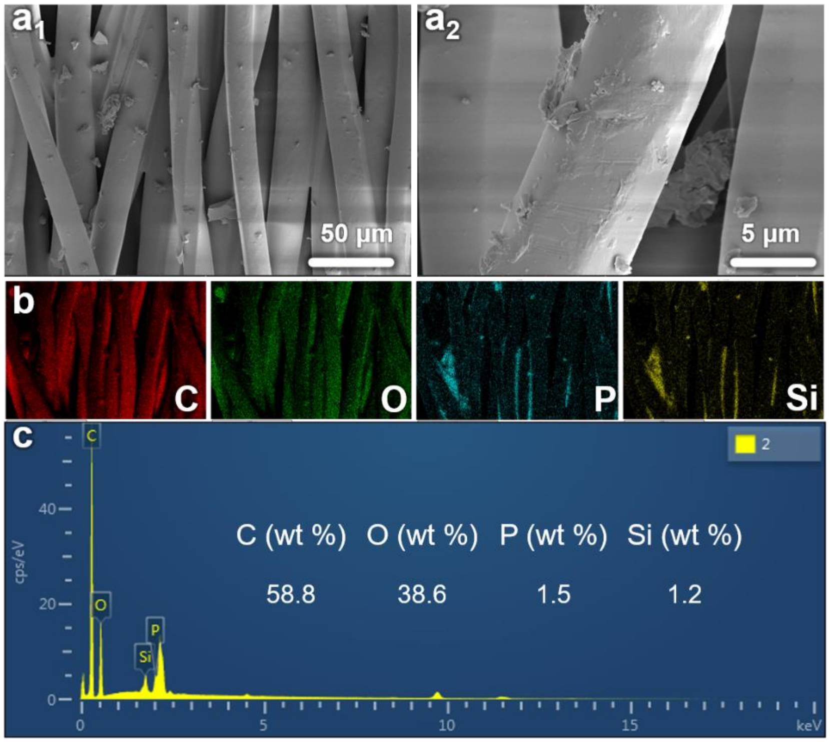

Fig. S26. Surface microstructure for coated PET after abrasion resistance test. (a_1, a_2) SEM micrographs and (b) C, O, P and Si element distribution maps for FRPET-3 after the abrasion resistance test. (c) EDS results and element contents for FRPET-3 after the abrasion resistance test.

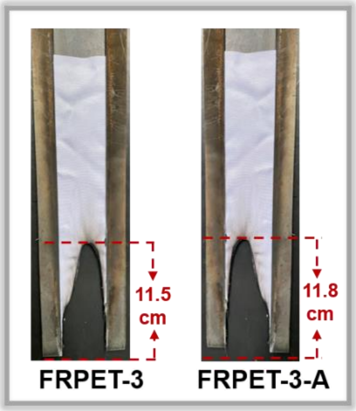

Fig. S27. Flame retardancy for coated PET after abrasion resistance test. Digital photographs after the vertical flame test for FRPET-3 against the abrasion resistance test for 50 cycles.

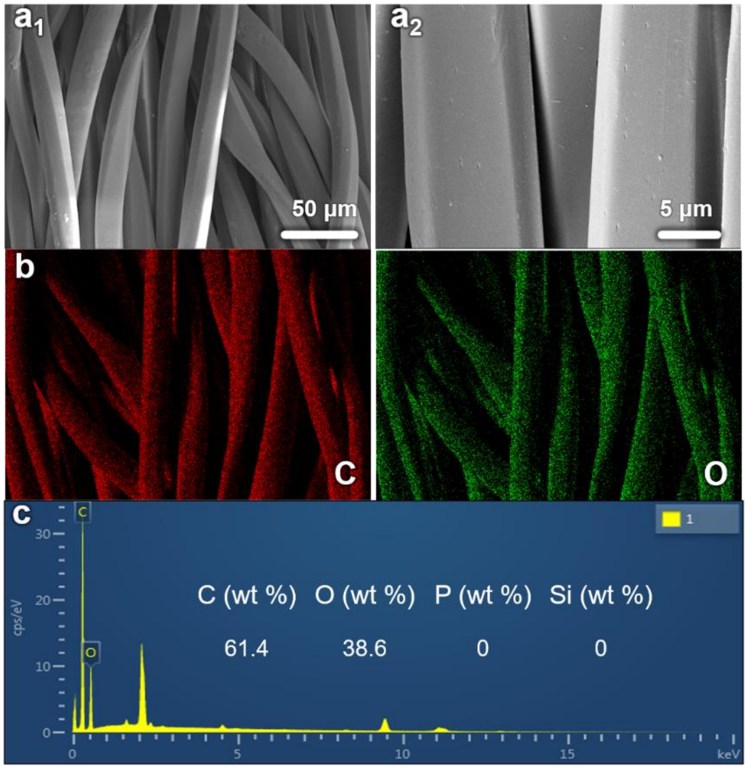

Fig. S28. Surface microstructure for coated PET after immersion in ethanol. (a_1, a_2) SEM micrographs and (b) C and O element distribution maps for FRPET-3 after soaking in excess ethanol. (c) EDS results and element contents for FRPET-3 after soaking in excess ethanol.

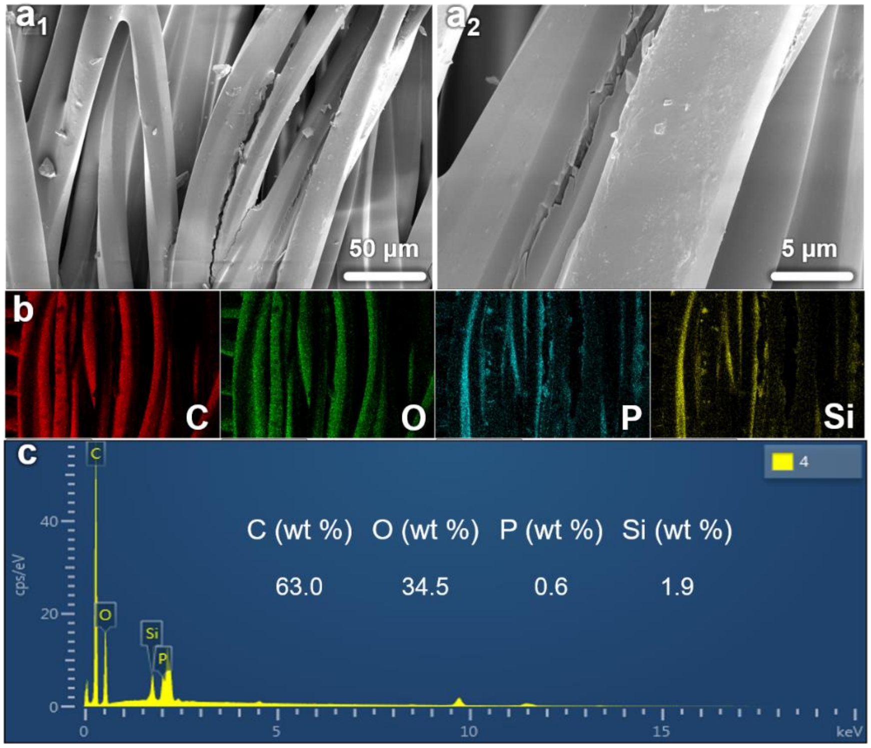

Fig. S29. Surface microstructure for recoated PET. (a_1, a_2) SEM micrographs and (b) C, O, P and Si element distribution maps for the recoated FRPET. (c) EDS results and element contents for the recoated FRPET.

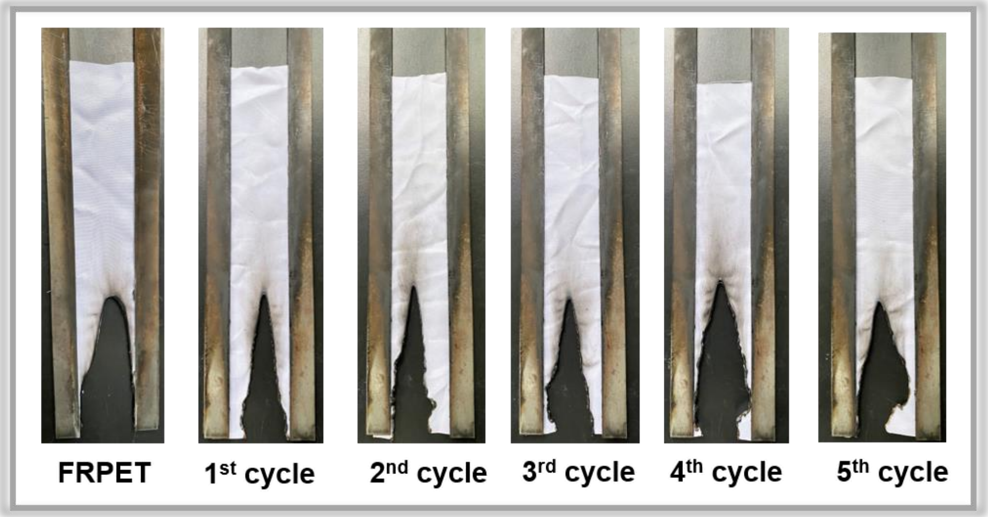

Fig. S30. Flame retardancy for recoated PET. Digital photographs after the vertical flame test for each recoated PET fabric.

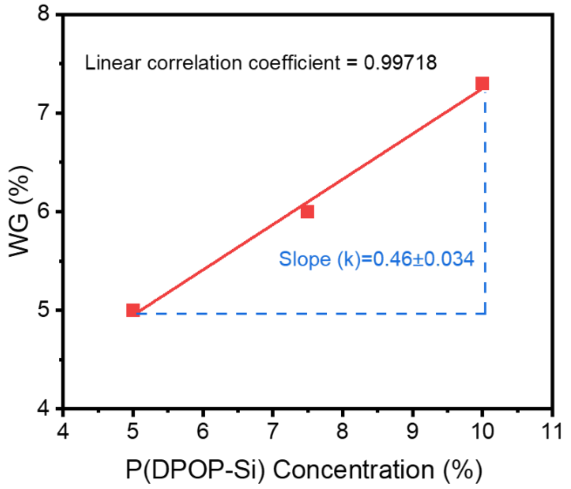

Fig. S31. Relationship between weight gain (WG) of the fabric and the precursor concentration of the coating.

Table S1. Formulations of FRPET fabrics with different weight gain (WG) of P(DPOP-Si) adhesive

Sample	P(DPOP-Si) (g)	Mixture medium (g)	Ethanol/H_2O (V/V)	Concentration (%)	WG (%)
FRPET-1	10	190	2/1	5.0	5.0
FRPET-2	15	185	2/1	7.5	6.0
FRPET-3	20	180	2/1	10.0	7.3

Table S2. Comparison of P(DPOP-Si) adhesive with other reported reversible adhesives in terms of shear adhesion strength, durability, transparency, reusability, and flame retardancy

Samples	Adhesion strength (MPa)	Transparency (%)	Haze (%)	Cycling numbers	Resistance	Flame retardancy	Refs
Our work	3.5	98	~2.5	100	High resistance to water, salt/acid/base solutions, and high/low temperatures	good	-
PDMS/graphene	0.02	-	-	50	Slight resistance to water	-	*(35)*
PU	0.1	96	-	100	-	-	*(36)*
PU-GSMP	0.3	-	-	6	-	-	*(23)*
Anth-PEI	0.6	-	-	3	Strong resistance to water	-	*(12)*
Ionogel adhesive	1.0	-	-	5	-	-	*(22)*
Azopolymer	1.2	-	-	4	Resistance to water	-	*(68)*
Azos	1.34	-	-	7	-	-	*(13)*
Photoresponsive LC	1.6	-	--	4	-	-	*(69)*
PC-Water adhesive	2.5	-	-	10	High resistance to low temperature (-80 °C)	-	*(70)*
IC gel	5.8	-	-	5	-	-	*(15)*
PEI-GA gel		97	-	-	-	-	*(71)*
HAEncap		90	-	-	-	-	*(38)*

Table S3. LOI values and VFT test results for the control PET and FRPET

Sample	WG (%)	LOI (%)	Vertical flame test			
			After-flame time (s)	After-glow time (s)	Damaged length (mm)	Dripping
Control PET	-	21.5±0.1	18	0	213±5	Yes
FRPET-1	5.0	25.9±0.1	9	0	149±6	No
FRPET-2	6.0	30.8±0.2	0	0	130±4	No
FRPET-3	7.3	34.5±0.1	0	0	115±7	No

Table S4. Cone calorimetry results for the control PET and FRPET

Sample	TTI (s)	T_{pHRR} (s)	pHRR (kW/m^2)	THR (MJ/m^2)	Reduction in pHRR (%)	Residue (%)	FIGRA ($kW/m^2\cdot s$)
Control PET	41	70	320.0±6	5.7±0.1	-	3.5	4.6
FRPET-1	52	80	303.4±5	4.2±0.1	5.2	7.0	3.8
FRPET-2	44	75	248.5±4	4.2±0.1	22.3	10.5	3.3
FRPET-3	40	75	223.3±5	4.2±0.1	30.2	10.9	3.0

Table S5. LOI values and VFT test results for PET, LFRPET and LFRPET-W

Sample	WG (%)	LOI (%)	Vertical flame test			
			After-flame time (s)	After-glow time (s)	Damaged length (mm)	Dripping
Control PET	-	21.5±0.1	18	0	213±5	Yes
FRPET-3 (Dip-coating)	7.3	34.5±0.1	0	0	115±7	No
LFRPET	11.3	34.5±0.1	0	0	100±5	No
LFRPET-W	11.0	-	0	0	105±3	No

Table S6. VFT test results for FRPET-3 after immersion in water for different days; LFRPET after immersion in water for 10 days and simulation machine washing for 50 cycles

Sample	Weight remaining rate (%)	Vertical flame test			
		After-flame time (s)	After-glow time (s)	Damaged length (mm)	Dripping
FRPET-3	-	0	0	115±3	No
2 days	97.3	0	0	110±6	No
4 days	96.8	0	0	112±5	No
6 days	96.8	0	0	114±3	No
8 days	96.8	0	0	118±1	No
10 days	96.8	0	0	117±1	No
LFRPET-W	97.0	0	0	105±3	No
LFRPET-MW	97.0	0	0	115±2	No

Table S7. VFT test results for FRPET-3 against the abrasion resistance test for 50 cycles

Sample	Weight remaining rate (%)	Vertical flame test			
		After-flame time (s)	After-glow time (s)	Damaged length (mm)	Dripping
FRPET-3	-	0	0	115±3	No
FRPET-3-A	98.7	0	0	118±1	No

Table S8. LOI values and VFT test results for each recoated PET fabric

Sample	Recycle rate (%)	LOI (%)	Vertical flame test			
			After-flame time (s)	After-glow time (s)	Damaged length (mm)	Dripping
FRPET	-	34.5±0.1	0	0	115±5	No
1st cycle	100	33.9±0.5	0	0	118±2	No
2nd cycle	100	34.3±0.2	0	0	115±6	No
3th cycle	100	34.5±0.1	0	0	123±1	No
4th cycle	100	34.1±0.3	0	0	125±1	No
5th cycle	100	34.0±0.4	0	0	115±7	No

Movie S1.
Macroscopic test of adhesion behavior for ethanol responsive.

Movie S2.
Combustion performance of control PET fabric.

Movie S3.
Combustion performance of coated PET fabric.

REFERENCES AND NOTES

1. W. Zhang, R. Wang, Z. Sun, X. Zhu, Q. Zhao, T. Zhang, A. Cholewinski, F. Yang, B. Zhao, R. Pinnaratip, P. K. Forooshani, B. P. Lee, Catechol-functionalized hydrogels: Biomimetic design, adhesion mechanism, and biomedical applications. *Chem. Soc. Rev.* **49**, 433–464 (2020).
2. J. Saiz-Poseu, J. Mancebo-Aracil, F. Nador, F. Busqué, D. Ruiz-Molina, The chemistry behind catechol-based adhesion. *Angew. Chem. Int. Ed.* **58**, 696–714 (2019).
3. R. Jinkins Katherine, S. Li, H. Arafa, H. Jeong, J. Lee Young, C. Wu, E. Campisi, X. Ni, D. Cho, Y. Huang, A. Rogers John, Thermally switchable, crystallizable oil and silicone composite adhesives for skin-interfaced wearable devices. *Sci. Adv.* **8**, eabo0537 (2022).
4. Y. Zhao, S. Song, X. Ren, J. Zhang, Q. Lin, Y. Zhao, Supramolecular adhesive hydrogels for tissue engineering applications. *Chem. Rev.* **122**, 5604–5640 (2022).
5. X. Ou, B. Xue, Y. Lao, Y. Wutthinitikornkit, R. Tian, A. Zou, L. Yang, W. Wang, Y. Cao, J. Li, Structure and sequence features of mussel adhesive protein lead to its salt-tolerant adhesion ability. *Sci. Adv*. **6**, eabb7620 (2020).
6. J. Luo, Y. Zhou, Q. Gao, J. Li, N. Yan, From wastes to functions: A new soybean meal and bark-based adhesive. *ACS Sustain. Chem. Eng.* **8**, 10767–10773 (2020).
7. C. A. Xu, Z. Qu, M. Lu, H. Meng, B. Chen, E. Jiao, E. Zhang, K. Wu, J. Shi, Effect of modified bamboo lignin replacing part of C5 petroleum resin on properties of polyurethane/polysiloxane pressure-sensitive adhesive and its application on the wood substrate. *J. Colloid Interface Sci.* **602**, 394–405 (2021).
8. Z. Jia, X. Feng, Y. Zou, Graphene reinforced epoxy adhesive for fracture resistance. *Compos. Part B Eng.* **155**, 457–462 (2018).
9. A. Kumar, A. J. Domb, Polymerization enhancers for cyanoacrylate skin adhesive. *Macromol. Biosci.* **21**, 2100143 (2021).
10. J. Han, Y. Guo, H. Wang, K. Zhang, D. Yang, Sustainable bioplastic made from biomass DNA and ionomers. *J. Am. Chem. Soc.* **143**, 19486–19497 (2021).
11. A. Beharaj, E. Z. McCaslin, W. A. Blessing, M. W. Grinstaff, Sustainable polycarbonate adhesives for dry and aqueous conditions with thermoresponsive properties. *Nat. Commun.* **10**, 5478 (2019).
12. Z. Wang, L. Guo, H. Xiao, H. Cong, S. Wang, A reversible underwater glue based on photo- and thermo-responsive dynamic covalent bonds. *Mater. Horiz.* **7**, 282–288 (2020).
13. Z. Wu, C. Ji, X. Zhao, Y. Han, K. Müllen, K. Pan, M. Yin, Green-light-triggered phase transition of azobenzene derivatives toward reversible adhesives. *J. Am. Chem. Soc.* **141**, 7385–7390 (2019).
14. L. M. de Espinosa, W. Meesorn, D. Moatsou, C. Weder, Bioinspired polymer systems with stimuli-responsive mechanical properties. *Chem. Rev.* **117**, 12851–12892 (2017).
15. L. Liu, Z. Liu, Y. Ren, X. Zou, W. Peng, W. Li, Y. Wu, S. Zheng, X. Wang, F. Yan, A superstrong and reversible ionic crystal-based adhesive inspired by ice adhesion. *Angew. Chem. Int. Ed.* **60**, 8948–8959 (2021).
16. J. Liu, C. S. Y. Tan, O. A. Scherman, Dynamic interfacial adhesion through cucurbit[n]uril molecular recognition. *Angew. Chem. Int. Ed.* **57**, 8854–8858 (2018).
17. W. Li, X. Liu, Z. Deng, Y. Chen, Q. Yu, W. Tang, T. L. Sun, Y. S. Zhang, K. Yue, Tough bonding, on-demand debonding, and facile rebonding between hydrogels and diverse metal surfaces. *Adv. Mater.* **31**, e1904732 (2019).

18. A. Rahman Md, C. Bowland, S. Ge, R. Acharya Shree, S. Kim, R. Cooper Valentino, X. C. Chen, S. Irle, P. Sokolov Alexei, A. Savara, T. Saito, Design of tough adhesive from commodity thermoplastics through dynamic crosslinking. *Sci. Adv.* **7**, eabk2451 (2021).
19. H. Cho, G. Wu, J. Christopher Jolly, N. Fortoul, Z. He, Y. Gao, A. Jagota, S. Yang, Intrinsically reversible superglues via shape adaptation inspired by snail epiphragm. *Proc. Natl. Acad. Sci. U.S.A.* **116**, 13774–13779 (2019).
20. Q. Zhang, T. Li, A. Duan, S. Dong, W. Zhao, P. J. Stang, Formation of a supramolecular polymeric adhesive via water–participant hydrogen bond formation. *J. Am. Chem. Soc.* **141**, 8058–8063 (2019).
21. S. Dong, J. Leng, Y. Feng, M. Liu, J. Stackhouse Chloe, A. Schönhals, L. Chiappisi, L. Gao, W. Chen, J. Shang, L. Jin, Z. Qi, A. Schalley Christoph, Structural water as an essential comonomer in supramolecular polymerization. *Sci. Adv.* **3**, eaao0900 (2017).
22. J. Zhu, X. Lu, W. Zhang, X. Liu, Substrate-independent, reversible, and easy-release ionogel adhesives with high bonding strength. *Macromol. Rapid Commun.* **41**, 2000098 (2020).
23. D. Tan, X. Wang, Q. Liu, K. Shi, B. Yang, S. Liu, Z.-S. Wu, L. Xue, Switchable adhesion of micropillar adhesive on rough surfaces. *Small* **15**, e1904248 (2019).
24. Y. Takashima, T. Sahara, T. Sekine, T. Kakuta, M. Nakahata, M. Otsubo, Y. Kobayashi, A. Harada, Supramolecular adhesives to hard surfaces: Adhesion between host hydrogels and guest glass substrates through molecular recognition. *Macromol. Rapid Commun.* **35**, 1646–1652 (2014).
25. L. Han, L. Yan, M. Wang, K. Wang, L. Fang, J. Zhou, J. Fang, F. Ren, X. Lu, Transparent, adhesive, and conductive hydrogel for soft bioelectronics based on light-transmitting polydopamine-doped polypyrrole nanofibrils. *Chem. Mater.* **30**, 5561–5572 (2018).
26. Y. Park, H. Byun, J. H. Lee, Highly stretchable and transparent optical adhesive films using hierarchically structured rigid-flexible dual-stiffness nanoparticles. *ACS Appl. Mater. Inter.* **13**, 1493–1502 (2021).
27. X. L. Wang, L. Chen, J. N. Wu, T. Fu, Y. Z. Wang, Flame-retardant pressure-sensitive adhesives derived from epoxidized soybean oil and phosphorus-containing dicarboxylic acids. *ACS Sustain. Chem. Eng.* **5**, 3353–3361 (2017).
28. M. Qiu, W. Du, X. Luo, S. Zhu, Y. Luo, J. Zhao, Vapor-phase molecular doping in covalent organosiloxane network thin films via a lewis acid–base interaction for enhanced mechanical properties. *ACS Appl. Mater. Inter.* **14**, 22719–22727 (2022).
29. R. M. Laine, J. C. Furgal, P. Doan, D. Pan, V. Popova, X. Zhang, Avoiding carbothermal reduction: Distillation of alkoxysilanes from biogenic, green, and sustainable sources. *Angew. Chem. Int. Ed.* **55**, 1065–1069 (2016).
30. X. Xun, Z. Zhang, X. Zhao, B. Zhao, F. Gao, Z. Kang, Q. Liao, Y. Zhang, Highly robust and self-powered electronic skin based on tough conductive self-healing elastomer. *ACS Nano* **14**, 9066–9072 (2020).
31. J. Kang, D. Son, G.-J. N. Wang, Y. Liu, J. Lopez, Y. Kim, J. Y. Oh, T. Katsumata, J. Mun, Y. Lee, L. Jin, J. B. H. Tok, Z. Bao, Tough and water-insensitive self-healing elastomer for robust electronic skin. *Adv. Mater.* **30**, 1706846 (2018).
32. M. Liu, Z. Wang, P. Liu, Z. Wang, H. Yao, X. Yao, Supramolecular silicone coating capable of strong substrate bonding, readily damage healing, and easy oil sliding. *Sci. Adv.* **5**, eaaw5643 (2019).

33. B. Yi, P. Liu, C. Hou, C. Cao, J. Zhang, H. Sun, X. Yao, Dual-cross-linked supramolecular polysiloxanes for mechanically tunable, damage-healable and oil-repellent polymeric coatings. *ACS Appl. Mater. Interfaces* **11**, 47382–47389 (2019).
34. H. Zhao, Q. Sun, X. Deng, J. Cui, Earthworm-inspired rough polymer coatings with self-replenishing lubrication for adaptive friction-reduction and antifouling surfaces. *Adv. Mater.* **30**, 1802141 (2018).
35. X. Wang, D. Tan, S. Hu, Q. Li, B. Yang, Z. Shi, R. Das, X. Xu, Z.-S. Wu, L. Xue, Reversible adhesion via light-regulated conformations of rubber chains. *ACS Appl. Mater. Inter.* **11**, 46337–46343 (2019).
36. M. Li, W. Li, Q. Guan, X. Dai, J. Lv, Z. Xia, W.-J. Ong, E. Saiz, X. Hou, A tough reversible biomimetic transparent adhesive tape with pressure-sensitive and wet-cleaning properties. *ACS Nano* **15**, 19194–19201 (2021).
37. X. Wang, W. Zhang, Z. Qin, R. Yang, Optically transparent and flame-retarded polycarbonate nanocomposite based on diphenylphosphine oxide-containing polyhedral oligomeric silsesquioxanes. *Compos. Part A Appl. S.* **117**, 92–102 (2019).
38. Y. H. Kim, Y.-W. Lim, D. Lee, Y. H. Kim, B.-S. Bae, A highly adhesive siloxane LED encapsulant optimized for high thermal stability and optical efficiency. *J. Mater. Chem. C* **4**, 10791–10796 (2016).
39. C. Jia, C. Chen, R. Mi, T. Li, J. Dai, Z. Yang, Y. Pei, S. He, H. Bian, S.-H. Jang, J. Y. Zhu, B. Yang, L. Hu, Clear wood toward high-performance building materials. *ACS Nano* **13**, 9993–10001 (2019).
40. Z. H. Zhao, P. C. Zhao, Y. Zhao, J. L. Zuo, C. H. Li, An underwater long-term strong adhesive based on boronic esters with enhanced hydrolytic stability. *Adv. Funct. Mater.* **32**, 2201959 (2022).
41. P. H. Wu, Y. Z. Lai, Y. P. Zhang, M. C. Sil, P.-H. H. Lee, T. C. Wei, C. M. Chen, Organosiloxane monolayers terminated with amine groups as adhesives for Si metallization. *ACS Appl. Nano Mater.* **3**, 3741–3749 (2020).
42. M. Cencer, Y. Liu, A. Winter, M. Murley, H. Meng, B. P. Lee, Effect of pH on the rate of curing and bioadhesive properties of dopamine functionalized poly(ethylene glycol) hydrogels. *Biomacromolecules* **15**, 2861–2869 (2014).
43. Y. Zhao, Y. Wu, L. Wang, M. Zhang, X. Chen, M. Liu, J. Fan, J. Liu, F. Zhou, Z. Wang, Bio-inspired reversible underwater adhesive. *Nat. Commun.* **8**, 2218 (2017).
44. S. Xi, F. Tian, G. Wei, X. He, Y. Shang, Y. Ju, W. Li, Q. Lu, Q. Wang, Reversible dendritic-crystal-reinforced polymer gel for bioinspired adaptable adhesive. *Adv. Mater.* **33**, e2103174 (2021).
45. H. Yan, P. Lee, N. R. Armstrong, A. Graham, G. A. Evmenenko, P. Dutta, T. J. Marks, High-performance hole-transport layers for polymer light-emitting diodes. Implementation of organosiloxane cross-linking chemistry in polymeric electroluminescent devices. *J. Am. Chem. Soc.* **127**, 3172–3183 (2005).
46. J. Jin, T. Iyoda, C. Cao, Y. Song, L. Jiang, T. J. Li, D. B. Zhu, Self-assembly of uniform spherical aggregates of magnetic nanoparticles through π–π interactions. *Angew. Chem. Int. Ed.* **40**, 2135–2138 (2001).
47. S. Wang, D. Bai, Y. Wang, J. Fu, J. Zhu, X. Fang, Hierarchical self-assembly of helical coordination polymers and formation of a lamellar structure via the cooperativity of two-step Ag(i) coordination and π–π interactions. *Nanoscale* **12**, 10972–10976 (2020).

48. Y. Song, Y. Liu, T. Qi, G. L. Li, Towards dynamic but supertough healable polymers through biomimetic hierarchical hydrogen-bonding interactions. *Angew. Chem. Int. Ed.* **57**, 13838–13842 (2018).
49. W. B. Ying, G. Wang, Z. Kong, C. K. Yao, Y. Wang, H. Hu, F. Li, C. Chen, Y. Tian, J. Zhang, R. Zhang, J. Zhu, A biologically muscle-inspired polyurethane with super-tough, thermal reparable and self-healing capabilities for stretchable electronics. *Adv. Funct. Mater.* **31**, 2009869 (2021).
50. X. Wang, S. Zhan, Z. Lu, J. Li, X. Yang, Y. Qiao, Y. Men, J. Sun, Healable, recyclable, and mechanically tough polyurethane elastomers with exceptional damage tolerance. *Adv. Mater.* **32**, e2005759 (2020).
51. X. Zhu, C. Wei, H. Chen, C. Zhang, H. Peng, D. Wang, J. Yuan, J. H. Waite, Q. Zhao, A cation-methylene-phenyl sequence encodes programmable poly(ionic liquid) coacervation and robust underwater adhesion. *Adv. Funct. Mater.* **32**, 2105464 (2022).
52. D. Wei, F. Ma, R. Wang, S. Dou, P. Cui, H. Huang, J. Ji, E. Jia, X. Jia, S. Sajid, A. M. Elseman, L. Chu, Y. Li, B. Jiang, J. Qiao, Y. Yuan, M. Li, Ion-migration inhibition by the cation–π interaction in perovskite materials for efficient and stable perovskite solar cells. *Adv. Mater.* **30**, 1707583 (2018).
53. Y. Wang, G. Xia, H. Yu, B. Qian, Y. H. Cheung, L. H. Wong, J. H. Xin, Mussel-inspired design of a self-adhesive agent for durable moisture management and bacterial inhibition on PET fabric. *Adv. Mater.* **33**, 2100140 (2021).
54. Z. Ma, X. Liu, X. Xu, L. Liu, B. Yu, C. Maluk, G. Huang, H. Wang, P. Song, Bioinspired, highly adhesive, nanostructured polymeric coatings for superhydrophobic fire-extinguishing thermal insulation foam. *ACS Nano* **15**, 11667–11680 (2021).
55. B. W. Liu, H. B. Zhao, Y. Z. Wang, Advanced flame-retardant methods for polymeric materials. *Adv. Mater.*, 2107905 (2021).
56. A. N. Zhang, H. B. Zhao, J. B. Cheng, M. E. Li, S. L. Li, M. Cao, Y. Z. Wang, Construction of durable eco-friendly biomass-based flame-retardant coating for cotton fabrics. *Chem. Eng. J.* **410**, 128361 (2021).
57. P. Song, C. Wang, L. Chen, Y. Zheng, L. Liu, Q. Wu, G. Huang, Y. Yu, H. Wang, Thermally stable, conductive and flame-retardant nylon 612 composites created by adding two-dimensional alumina platelets. *Compos. Part A Appl. Sci.* **97**, 100–110 (2017).
58. W. Rao, J. Shi, C. Yu, H. B. Zhao, Y. Z. Wang, Highly efficient, transparent, and environment-friendly flame-retardant coating for cotton fabric. *Chem. Eng. J.* **424**, 130556 (2021).
59. G. Loke, W. Yan, T. Khudiyev, G. Noel, Y. Fink, Recent progress and perspectives of thermally drawn multimaterial fiber electronics. *Adv. Mater.* **32**, e1904911 (2020).
60. W. Yan, G. Noel, G. Loke, E. Meiklejohn, T. Khudiyev, J. Marion, G. Rui, J. Lin, J. Cherston, A. Sahasrabudhe, J. Wilbert, I. Wicaksono, R. W. Hoyt, A. Missakian, L. Zhu, C. Ma, J. Joannopoulos, Y. Fink, Single fibre enables acoustic fabrics via nanometre-scale vibrations. *Nature* **603**, 616–623 (2022).
61. A. Sadezky, H. Muckenhuber, H. Grothe, R. Niessner, U. Pöschl, Raman microspectroscopy of soot and related carbonaceous materials: Spectral analysis and structural information. *Carbon* **43**, 1731–1742 (2005).
62. T. Fu, D. M. Guo, L. Chen, W. S. Wu, X. L. Wang, Y. Z. Wang, Fire hazards management for polymeric materials via synergy effects of pyrolysates-fixation and aromatized-charring. *J. Hazard. Mater.* **389**, e122040 (2020).

63. J. Schwan, S. Ulrich, V. Batori, H. Ehrhardt, S. R. P. Silva, Raman spectroscopy on amorphous carbon films. *J. Appl. Phys.* **80**, 440–447 (1996).
64. X. Wang, Y. Hu, L. Song, W. Xing, H. Lu, P. Lv, G. Jie, Flame retardancy and thermal degradation mechanism of epoxy resin composites based on a DOPO substituted organophosphorus oligomer. *Polymer* **51**, 2435–2445 (2010).
65. J. Vasiljević, I. Jerman, G. Jakša, J. Alongi, G. Malucelli, M. Zorko, B. Tomšič, B. Simončič, Functionalization of cellulose fibres with DOPO-polysilsesquioxane flame retardant nanocoating. *Cellulose* **22**, 1893–1910 (2015).
66. Z. Li, M. Chen, S. Li, X. Fan, C. Liu, Simultaneously improving the thermal, flame-retardant and mechanical properties of epoxy resins modified by a novel multi-element synergistic flame retardant. *Macromol. Mater. Eng.* **304**, e1800619 (2019).
67. B. W. Liu, L. Chen, D. M. Guo, X. F. Liu, Y. F. Lei, X. M. Ding, Y. Z. Wang, Fire-safe polyesters enabled by end-group capturing chemistry. *Angew. Chem. Int. Ed.* **58**, 9188–9193 (2019).
68. Y. Zhou, M. Chen, Q. Ban, Z. Zhang, S. Shuang, K. Koynov, H.-J. Butt, J. Kong, S. Wu, Light-switchable polymer adhesive based on photoinduced reversible solid-to-liquid transitions. *ACS Macro Lett.* **8**, 968–972 (2019).
69. S. Saito, S. Nobusue, E. Tsuzaka, C. Yuan, C. Mori, M. Hara, T. Seki, C. Camacho, S. Irle, S. Yamaguchi, Light-melt adhesive based on dynamic carbon frameworks in a columnar liquid-crystal phase. *Nat. Commun.* **7**, 12094 (2016).
70. X. Li, J. Lai, Y. Deng, J. Song, G. Zhao, S. Dong, Supramolecular adhesion at extremely low temperatures: A combined experimental and theoretical investigation. *J. Am. Chem. Soc.* **142**, 21522–21529 (2020).
71. Y. M. Seo, W. Jang, T. Gu, H. J. Seok, S. Han, B. L. Choi, H. K. Kim, H. Chae, J. Kang, D. Whang, Defect-free mechanical graphene transfer using *n*-doping adhesive gel buffer. *ACS Nano* **15**, 11276–11284 (2021).
